# 14-3-3ε Mediates the Cell Fate Decision-Making Pathways in Response of Hepatocellular Carcinoma to Bleomycin-Induced DNA Damage

**DOI:** 10.1371/journal.pone.0055268

**Published:** 2013-03-05

**Authors:** Siwei Tang, Huimin Bao, Yang Zhang, Jun Yao, Pengyuan Yang, Xian Chen

**Affiliations:** 1 Department of Chemistry and Institutes of Biomedical Sciences, Fudan University, Shanghai, China; 2 Department of Biochemistry and Biophysics, University of North Carolina at Chapel Hill, Chapel Hill, North Carolina, United States of America; Moffitt Cancer Center, United States of America

## Abstract

**Background:**

Lack of understanding of the response of hepatocellular carcinoma (HCC) to anticancer drugs causes the high mortality of HCC patients. Bleomycin (BLM) that induces DNA damage is clinically used for cancer therapy, while the mechanism underlying BLM-induced DNA damage response (DDR) in HCC cells remains ambiguous. Given that 14-3-3 proteins are broadly involved in regulation of diverse biological processes (BPs)/pathways, we investigate how a 14-3-3 isoform coordinates particular BPs/pathways in BLM-induced DDR in HCC.

**Methodology/Principal Findings:**

Using dual-tagging quantitative proteomic approach, we dissected the 14-3-3ε interactome formed during BLM-induced DDR, which revealed that 14-3-3ε *via* its associations with multiple pathway-specific proteins coordinates multiple pathways including chromosome remodeling, DNA/RNA binding/processing, DNA repair, protein ubiquitination/degradation, cell cycle arrest, signal transduction and apoptosis. Further, “zoom-in” investigation of the 14-3-3ε interacting network indicated that the BLM-induced interaction between 14-3-3ε and a MAP kinase TAK1 plays a critical role in determining cell propensity of apoptosis. Functional characterization of this interaction further revealed that BLM triggers site-specific phosphorylations in the kinase domain of TAK1. These BLM-induced changes of phosphorylations directly correlate to the strength of the TAK1 binding to 14-3-3ε, which govern the phosphorylation-dependent TAK1 activation. The enhanced 14-3-3ε-TAK1 association then inhibits the anti-apoptotic activity of TAK1, which ultimately promotes BLM-induced apoptosis in HCC cells. In a data-dependent manner, we then derived a mechanistic model where 14-3-3ε plays the pivotal role in integrating diverse biological pathways for cellular DDR to BLM in HCC.

**Conclusions:**

Our data demonstrated on a systems view that 14-3-3ε coordinates multiple biological pathways involved in BLM-induced DDR in HCC cells. Specifically, 14-3-3ε associates with TAK1 in a phosphorylation-dependent manner to determine the cell fate of BLM-treated HCC cells. Not only individual proteins but also those critical links in the network could be the potential targets for BLM-mediated therapeutic intervention of HCC.

## Introduction

Hepatocellular carcinoma (HCC) is the third leading cause of cancer death worldwide with a high recurrence and therefore an extremely low 5-year survival rate [1]. The high mortality rate is mainly due to unknown mechanisms of HCC pathogenesis as well as lack of strategies for HCC-specific therapeutic intervention. Bleomycin (BLM), a glycopeptide drug originally isolated from *Streptomyces verticillus* [2], is clinically used for cancer therapy [3], [4]. Once activated, BLM induces DNA damage, primarily double-stranded DNA breaks (DSBs) similar to those generated by ionizing radiation [5]. Meanwhile, cellular responses to BLM-induced DNA damage/DSBs are highly complex and are different from one cell type to another, leading to different outcomes or cell fates including cell cycle arrest or apoptosis/cell death or survival with DNA repair [5]. To develop more precise and effective strategies for HCC therapy, the mechanistic details regarding the drug-induced DNA damage response (DDR) of HCC cells need to be revealed.

14-3-3 proteins, the small, acidic, and abundant proteins with propensity to form both homo- and hetero-dimers, belong to a family of conserved regulatory molecules expressed in all eukaryotic cells [6], [7]. In mammals, seven isoforms including 14-3-3β, γ, ε, η, σ, θ (or τ) and ζ are known to interact with a broad range of the human proteome including numerous signaling and proto-oncogene proteins [8]–[10]. Through interacting with close to 300 target proteins identified so far, these 14-3-3 isomers are known to be involved in widespread biological processes such as signal transduction, cell cycle control, apoptosis, cellular metabolism, proliferation, cytoskeletal regulation, transcription, and redox-regulation or stress response, *etc* [6]–[8], [11], [12]. Diverse 14-3-3 interacting proteins include protein kinases, receptors, enzymes, structural and cytoskeletal proteins, scaffold molecules, DNA binding proteins and regulatory proteins [13]. 14-3-3 proteins exert their biological roles often by binding to specific phosphorylated sites in target proteins [7], [8]. By broadly interacting with over 200 human phosphoproteins, 14-3-3 proteins are central to integrate the regulation of biosynthetic metabolism, cell proliferation, survival and trafficking [14]. They also are involved in regulating the subcellular distribution of many phosphorylated targets, dynamic nucleocytoplasmic shuttling and protecting target proteins from modifications [15].

Further, emerging data of the identified isoform-specific interactions has suggested that an individual isoform probably has its specific biological roles [16], [17]. For example, 14-3-3σ has been specifically linked to cancer development, and the silencing of 14-3-3σ expression by CpG methylation is common during carcinogenesis [18]. So far, many known binding proteins have been reported to interact with 14-3-3σ, β, γ, θ, and ζ isoforms respectively [17], [19], [20]. 14-3-3ε isoform is the most conserved member of 14-3-3 family, with conserved sequence from plant, yeast and mammals [21], [22]. More importantly, the 14-3-3ε gene has been proposed to be a candidate tumor suppressor gene [6], [23], and it was also found to be involved in regulating carcinogenesis through differential expression proteome analysis in various HCC cell lines [24], [25]. In addition, the 14-3-3ε protein has been identified in lung cancer and it may be involved in lung carcinogenesis [26].

Previously, several reports identified a few putative proteins associated with 14-3-3ε through yeast two-hybrid approach or immunoprecipitation. For example, 14-3-3ε was found to interact with human calmodulin in a yeast two-hybrid approach, and their association may regulate cell signal transduction and cell proliferation [27]. 14-3-3ε also forms heterodimers with 14-3-3β, γ, η and ζ [22], [28]. Given the diverse roles of 14-3-3ε in carcinogenesis, we hypothesize that 14-3-3ε may broadly coordinate multiple biological processes (BPs)/pathways in cellular DNA damage response (DDR), which can be revealed by a systemic dissection of phenotypic 14-3-3ε protein-interacting network (interactome) assembled in HCC cells. We have previously demonstrated the use of quantitative proteomics to dissect HCC-specific 14-3-3ε interactome [29]. Here using the similar proteomic approach for screening a HCC-specific interactome [30], we have first dissected and identified putative composites of the 14-3-3ε interactome formed in HCC cells during cellular response to BLM treatment. Data-dependent network analysis allows us to map out the functional links between 14-3-3ε and many putative interacting proteins involved in multiple pathways and biological processes (BPs) responsible for BLM-induced DDR in HCC cells. Also “zoom-in” characterization of a core interaction between 14-3-3ε and a mitogen-activated protein kinase (MAPK), TAK1, provides an insight into the functional role of 14-3-3ε in modulating the activity of multiple pathway modules for BLM-induced cell fate decisions.

## Results

### Dissection of the BLM-induced 14-3-3ε interactome using dual-tagging quantitative proteomic approach

Similar to what previously described [30], we first generated a HCC cell line stably expressing the FLAG-tagged human 14-3-3ε, in which a FLAG tag is fused immediately at the N-terminus of 14-3-3ε. The expression level of exogenous FLAG-tagged 14-3-3ε was shown close to or even less than that of endogenous 14-3-3ε (Figure S1A), ensuring the physiological relevance of the pulled-down 14-3-3ε interactome for our study. Meanwhile, in line with the previous observations that 14-3-3ε forms homo- or hetero-dimers with selected isoforms of 14-3-3 for its function [31], FLAG-tagged 14-3-3ε was found co-immunoprecipitated with 14-3-3ζ, one of the isoforms in the 14-3-3 family (Figure S1B), all indicating that the FLAG tag fused on the N-terminus of 14-3-3ε does not affect the structure and function of 14-3-3ε. Furthermore, these stable HCC cells showed a growth rate and morphology similar to those of their parental cells, indicating that the expression of the FLAG-tagged 14-3-3ε has no effect on the phenotype of the stable cells.

**Figure 1:**
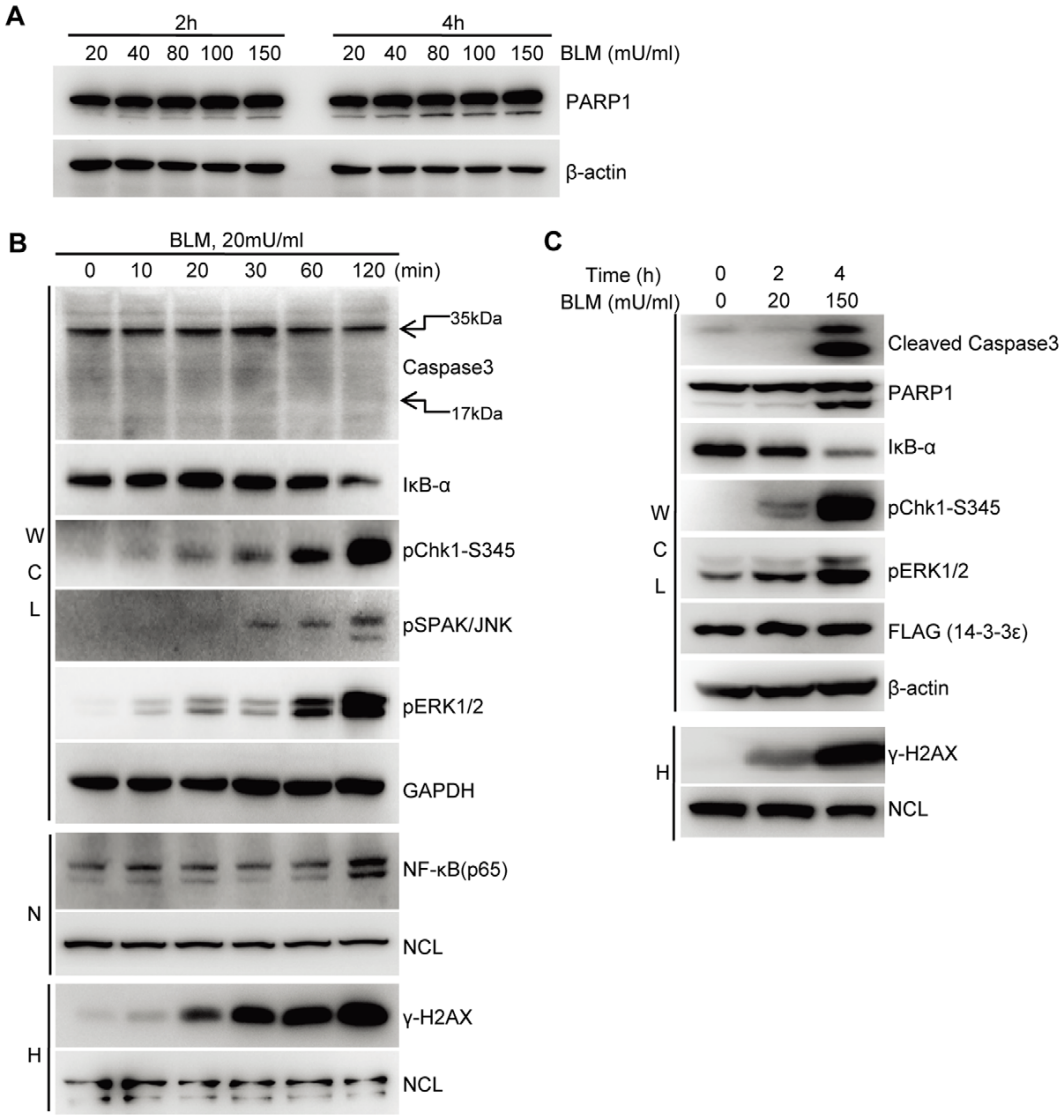
BLM triggers the activation of multiple signaling pathways in the HCC cells stably expressing FLAG-tagged 14-3-3ε. **A.** Immunoblotting analysis of dose-dependent changes of BLM-induced HCC propensity towards apoptosis with cleaved PARP1 as the apoptotic marker. **B.** Immunoblotting analysis of time-dependent changes of indicated markers induced by 20 mU/ml BLM. **C.** Combined dose- and time-dependent effect of BLM stimulation on the DDR of HCC cells. WCL: whole cell lysates; N: nuclear fraction; H: HCl extracted fraction. NCL was used as the loading control for nuclear and HCl-extracted fractions.

Given that BLM is a DNA damage response (DDR) inducer, we first examined both dose- and time-dependent response of the 14-3-3ε stable HCC cells to BLM to determine the maximum DDR, where the DDR-associated composition of the 14-3-3ε interactome in BLM-treated HCC cells was profiled/determined. First, we evaluated the dose-dependent BLM effect on the HCC cells collected at 2 and 4 hours following BLM treatment, respectively. As the results shown in Figure 1A, BLM induced HCC cells to undergo apoptosis in a dose- and time-dependent manner. HCC cells treated with 150 mU/ml BLM for 4 h showed apparent apoptosis reflected by the extent of the cleavage of PARP1, which is a marker indicative of the cellular status of apoptosis [32], while treatment with 20 mU/ml for 2 h led to much less apoptotic cells.

Following treatment of HCC with 20 mU/ml BLM for different time points, as results shown in Figure 1B, both levels of phosphoserine 139-(γ)-H2AX and phosphorylated Chk1 at serine 345 (pS345), the commonly used DNA damage markers [33], [34], were increased in a BLM-inducible and time-dependent manner and were reached to activation peak at 2h time point. Several MAPKs such as ERK1/2 and SPAK/JNK (p54/p46) were also induced by BLM in a similar way. Given that MAPK activation often results in the phosphorylation-dependent IκB-α degradation and concurrent translocation of NF-κB (p65) to nuclei, we observed that both IκB-α degradation and the nuclear entry of p65 were also reached to a peak at 2 h time point, all indicating the full activation of DDR signaling. Further, based on that activation of Caspase-3 that is an executioner of apoptosis requires a proteolytic process leading to activated p17 and p12 fragments [35], [36], no release of activated p17 fragment was detected under the same condition. These results together indicated that a 2 h treatment by BLM at 20 mU/ml could trigger a strong DDR in the HCC stable cells with a minimum apoptosis, which while a longer BLM treatment, *e.g.,* 4 hours, could promote elevated DDR but with apoptosis (Figure 1A). Meanwhile, we also checked the stable HCC cells treated with a high-dose BLM (150 mU/ml) for 4 h. Although DDR was further elevated compared to that of cells treated with low-dose BLM (20 mU/ml) for 2 h, the cells clearly underwent apoptosis as indicated by cleavage of Caspase 3 and its substrate, PARP1 (Figure 1C). Therefore, HCC cells treated with 20mU/ml for 2 h were chosen as the condition for profiling BLM-induced HCC-specific 14-3-3ε interactome using quantitative proteomic approach.

**Figure 2:**
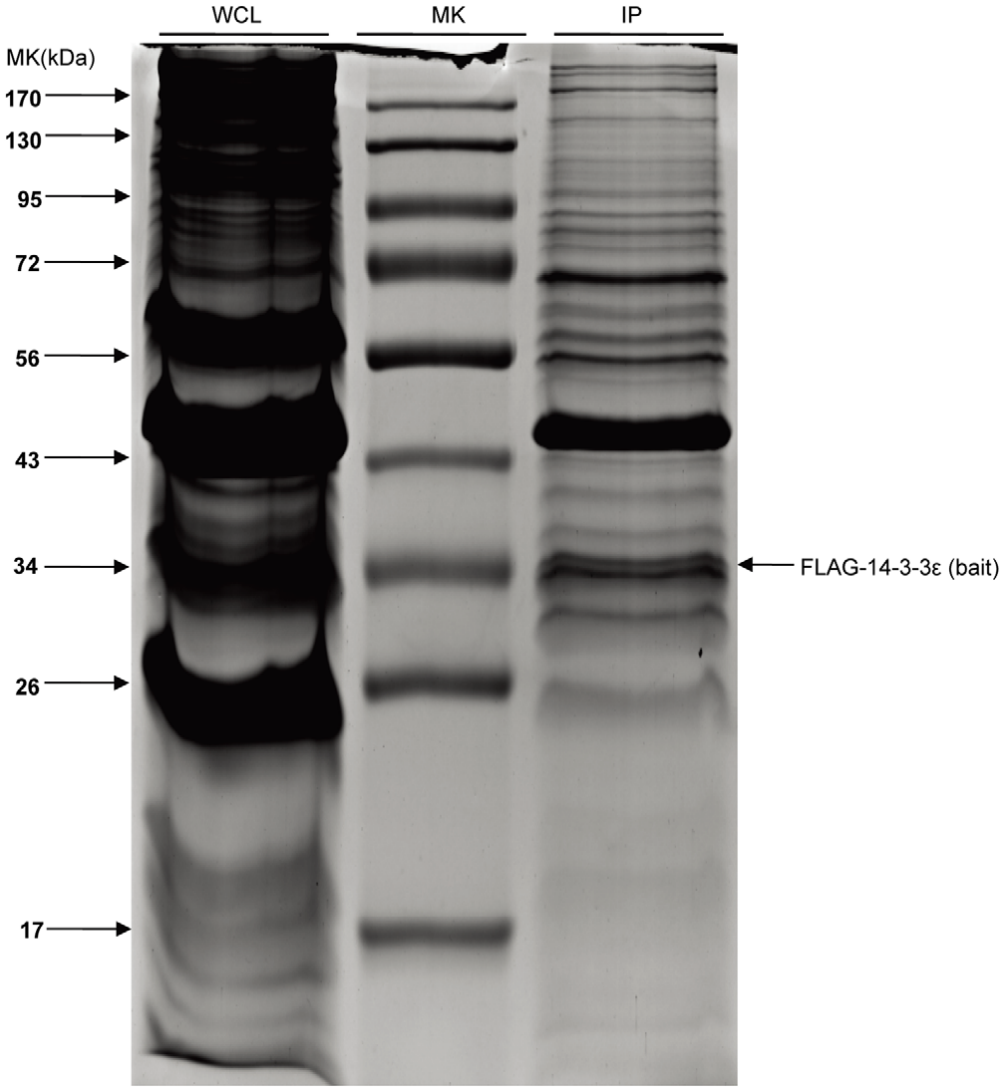
Isolation of 14-3-3ε complex by immunoprecipitation. Purification of 14-3-3ε complex was carried out via the procedure described in Materials and Methods. Proteins were separated on SDS-PAGE and stained with Coomassie Blue. WCL: whole cell lysates; IP: immunoprecipitates; MK: protein marker. Bait protein FLAG-tagged 14-3-3ε is indicated by arrow on SDS-PAGE gel.

Concurrently, in our quantitative proteomic design for interactome dissection, similar to what previously described [37] (Figure S2), the stable HCC cells maintained in the “Light” (L) medium were subjected to a stimulation of BLM at 20 mU/ml for 2 h, whereas the non-stimulated cells were grown in the “heavy” (H) medium containing leucine-d_3_. The 14-3-3ε immunoprecipitate from each cell pool was pulled-down using anti-FLAG beads respectively, and were mixed at ratio 1:1 based on the total protein mass followed by SDS-PAGE separation (Figure 2), in-gel trypsin digestion, and nano-LC-MS/MS analysis. Note that the post-immunoprecipitation mixing scheme was used to avoid possible light-to-heavy exchanges of the protein mixture [38]. Using the statistics criteria previously described [37], [39], by averaging the relative standard deviation (RSD) of all quantified proteins that have multiple peptides (≥2) containing leucine (in total 432 proteins), we found that the RSDs in all quantifiable proteins were approximately 12%. We therefore conservatively considered three times of the average RSD or greater than 36% as the threshold for distinguishing BLM-induced 14-3-3ε interacting proteins, *i.e.,* when the abundance of individual proteins around the bait 14-3-3ε is enriched by 40% or L/H≥1.40 in the immunoprecipitate pulled down from BLM-treated cells, it could be considered as a putative 14-3-3ε interactor. This cutoff value (40% in abundance increases) was further validated based on the protein ratio variability of all quantifiable proteins, which were calculated by the latest version of Proteome Discoverer software (v1.3, Thermo Scientific) with a ‘SILAC Quantitation’ module (described in detail in the Material and Methods and Figure S3). Also, the AACT/SILAC ratio of 14-3-3ε was found at L/H = 0.97, and the L/H ratio of 14-3-3ε (bait protein) was found close to 1:1(Figure S4A), indicating the accuracy of our mixing of the immunoprecipitates. We therefore normalized the ratios of all quantifiable proteins based on that of 14-3-3ε.

According to the quantitative criteria described above, among a total of 953 protein identifications 769 of them were quantified with normalized ratios (Table S1–S2). Therefore, those proteins showing L/H ratios less than 1.40 were considered as either non-specific associations or disassociations from 14-3-3ε, or constitutive 14-3-3ε interactors such as other 14-3-3 isoforms (Table S2). Meanwhile, 160 proteins were identified with their L/H ratios greater than 1.40. We also noted that there were some highly abundant proteins showing large L/H ratios, probably due to their stickiness to the agarose beads where FLAG antibody is conjugated to. Based on what previously reported [40], they are in the class of ‘bead proteome’. After excluding these proteins we identified 65 proteins with L/H ratios >1.40 as putative BLM-induced HCC-specific 14-3-3ε interactors (Table S2–S3).

### 14-3-3ε is a regulator broadly coordinating multiple cell fate-determining pathways involved in the programme of BLM-induced DDR

**Figure 3:**
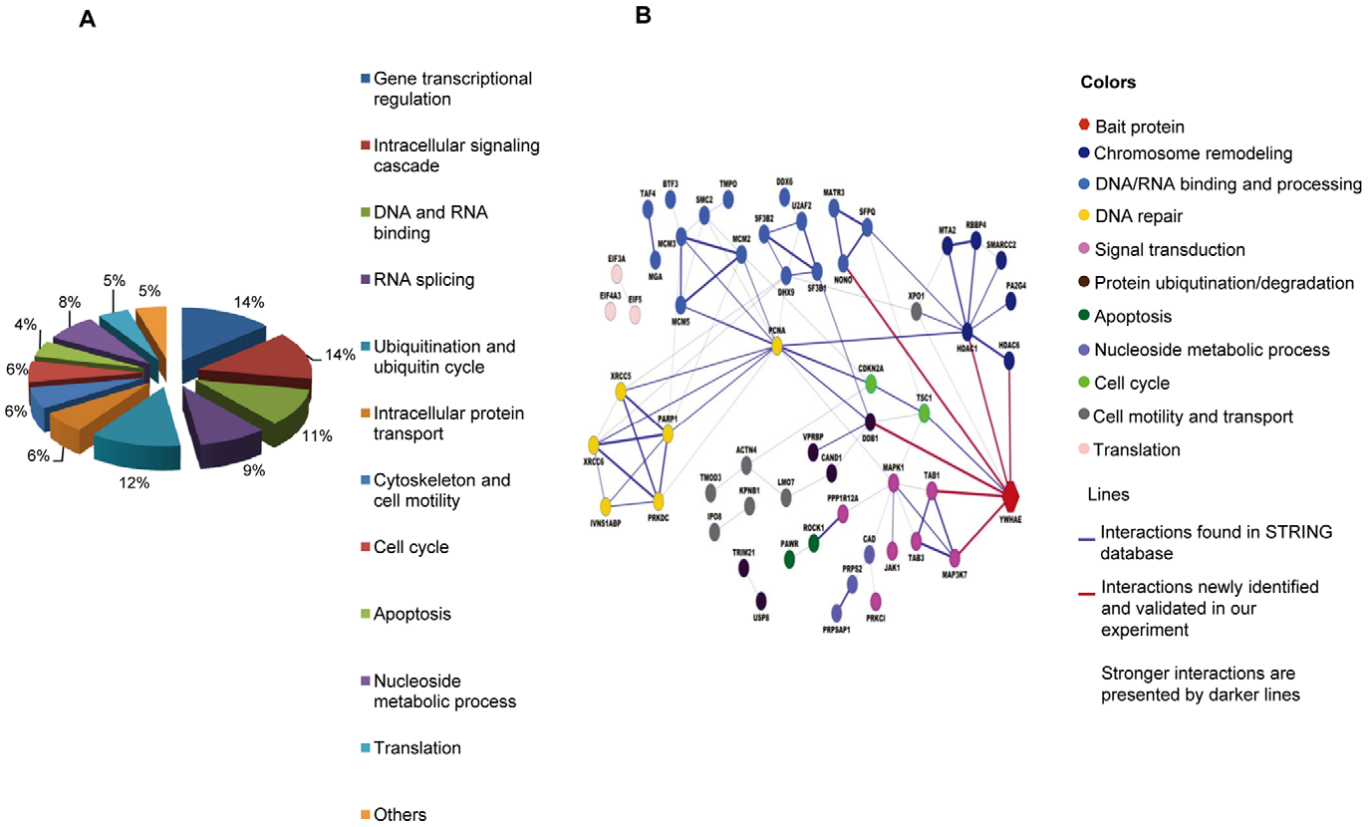
Functional categorization and network analysis of BLM-induced HCC-specific 14-3-3ε interactome. **A.** Functional categories and the corresponding percentage of the BLM-induced 14-3-3ε interacting partners identified by dual-tagging proteomic approach. The BLM-induced HCC-specific 14-3-3ε interactors were categorized according to their known involvements in various biological processes (BPs) by using online bioinformatics analysis tools such as Gene Ontology (http://www.geneontology.org/) and DAVID (http://www.david.abcc.ncifcrf.gov), and then the corresponding percentage of those interactors involved in different BPs were plotted. **B.** Data-dependent network analysis of 14-3-3ε interactors identified in the BLM-stimulated HCC cells. The gene symbol of 14-3-3ε is YWHAE. The network was constructed by the bioinformatics analysis tool: STRING (http://string.embl.de/). The links in the network were edited by Cytoscape 2.6 (http://www.cytoscape.org/).

We explored the functional relevance of these BLM-inducible HCC-specific 14-3-3ε interactors identified by our proteomic approach. Using Gene Ontology (http://www.geneontology.org/) and DAVID (http://www.david.abcc.ncifcrf.gov) to search for their known functions, the BLM-induced 14-3-3ε interactors were found in multiple functional categories/biological processes (BPs) such as gene transcriptional regulation (14%), signal transduction (14%), DNA/RNA binding and splicing (20%), and protein degradation (12%) along with protein transport, cytoskeleton/cell motility, cell cycle, apoptosis, nucleoside metabolic process and protein translation (Figure 3A). each of BLM-induced 14-3-3ε interactors that falls into different BPs was summarized and listed in Table S3.

We then biologically validated the physiological accuracy of newly MS-identified BLM-inducible 14-3-3ε interactors representing different BPs/pathways. The representative candidates of 14-3-3ε interactors include HDAC1, HDAC6, NONO, DDB1, TAK1 and TAB1. Their known relevance to cellular DDR is briefly described: Histone deacetylases (HDACs) such as HDAC1 and HDAC6 are known to be associated with other factors such as RBBP4, MTA2 and SMARCC2 to form macroprotein complexes that provide epigenetic control of gene expression [41]–[43]. Furthermore, a recent work revealed that both HDAC1 and HDAC2 were recruited to DNA-damage sites to regulate site-specific acetylation on histone H3 for promoting DSB signaling and DNA repair, particularly through non-homologous end-joining (NHEJ) pathway [44]. NONO, a ubiquitously expressed proteins originally identified as a non-POU domain containing and octamer-binding protein, possesses both DNA and RNA binding domains. NONO was shown to enhance the binding of many DNA-binding proteins at their recognition sites, implying that NONO may play a crucial role in regulation of gene expression [45]. NONO via its associations with SFPQ (Splicing factor proline/glutamine-rich) and MATR3 (Matrin 3) is directly involved in the NHEJ machinery (*e.g.,* Ku70/Ku80) to promote DSBs repair [46]. Damage-specific DNA binding protein 1(DDB1) is a key factor of CUL4-ROC1 ubiquitin E3 ligases [47]. The DDB1-CUL4-ROC1 system exerts multiple functions in cellular DDR. For instance, DDB1-CUL4 targets cyclinE and p27^Kip1^ to cause cell cycle arrest [48], and assembles with WDR5 and RBBP5 to regulate histone methylation, and therefore epigenetic control of gene transcription [49].

**Figure 4:**
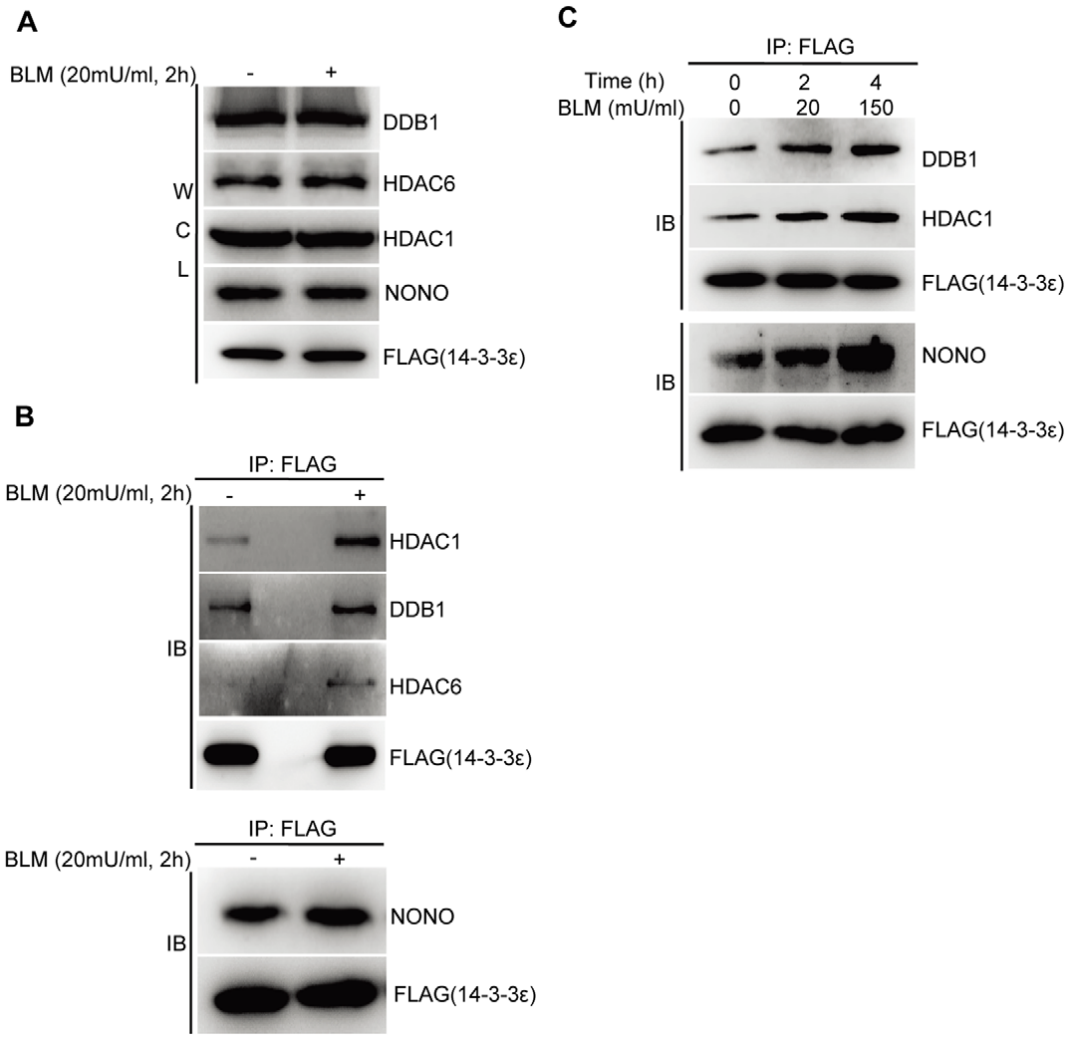
14-3-3ε is a pivotal regulator involving in BLM-induced DDR in HCC cells. **A–B.** Immunoblotting analysis of MS-identified 14-3-3ε interactors including HDAC1, DDB1, HDAC6 and NONO. The immunoprecipitates were similarly obtained from the stable 14-3-3ε HCC cells by using anti-FLAG beads before immunoblotting against indicated antibodies. **C.** Immunoblotting analysis of BLM-induced time-dependent 14-3-3ε interactions with some BPs-specific factors such as HDAC1, NONO and DDB1.

**Figure 5:**
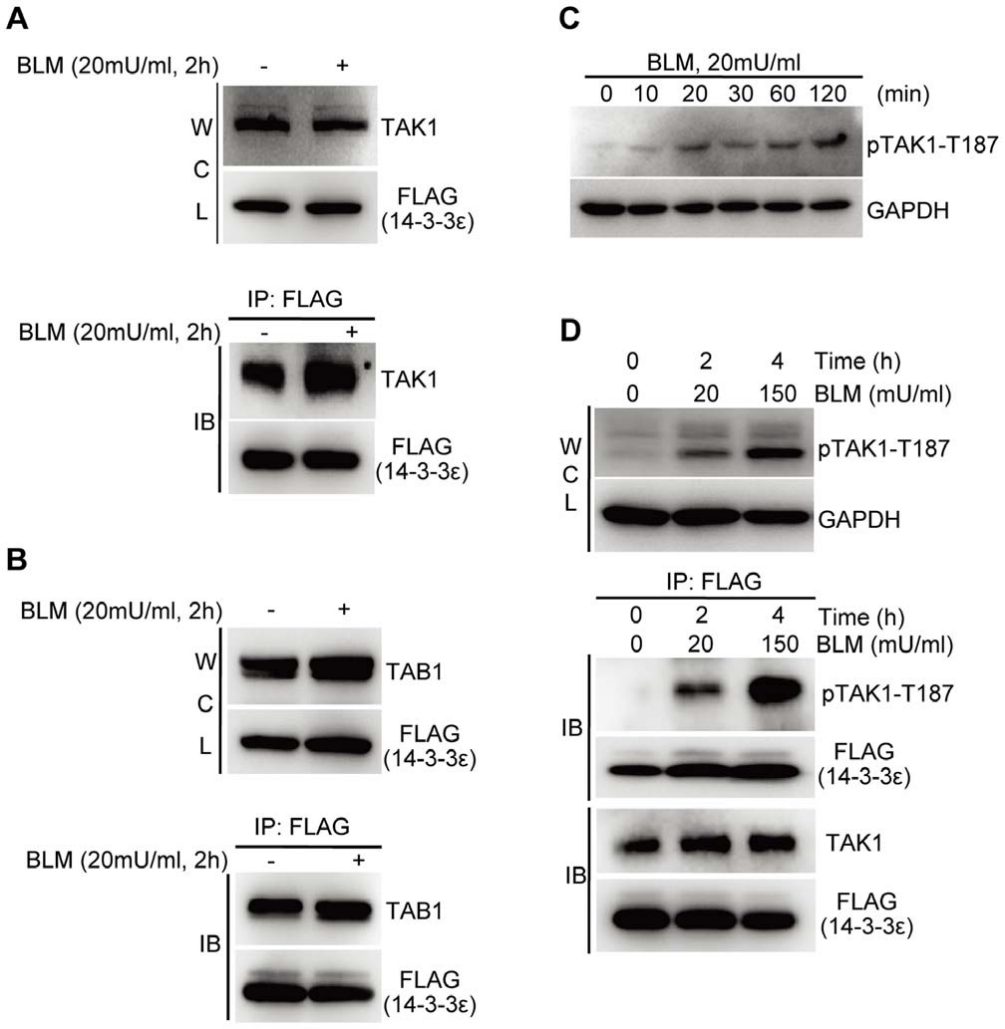
BLM-triggered association between 14-3-3ε and TAK1 correlates to the phosphorylation-dependent activation of TAK1. **A.** Immunoblotting analysis of the BLM-induced interaction between 14-3-3ε and TAK1. **B.** Immunoblotting analysis of the BLM-induced interaction between 14-3-3ε and the known TAK1 binding protein TAB1. **C.** BLM-induced time-dependent phosphorylation changes of TAK1in HCC cells. The whole cell lysates were blotted against anti-phospho-TAK1 (Thr187) antibody. **D.** The change of phosphorylated TAK1 interacting with 14-3-3ε of HCC response to dose-dependent BLM stimulation.

By performing co-immunoprecipitation/immunoblotting experiments, proteins such as HDAC1, HDAC6, NONO, DDB1, TAK1 and TAB1 were indeed shown to be BLM-induced 14-3-3ε interactors (Figure 4A-B, Figure 5A-B and Figure S4-S5). Further, we also clarified how the dynamics of these 14-3-3ε interactions correlates with the BLM-stimulated HCC cells with different apoptotic propensity. The stable HCC cells were also stimulated with a high-dose BLM at 150 mU/ml for 4 h where the status of HCC apoptosis was marked by the cleavage of Caspase 3 and its substrate, PARP1(Figure 1C). As the results shown in Figure 4C, the binding between 14-3-3ε and each of these BP/pathway-specific protein factors such as HDAC1, DDB1 and NONO was significantly enhanced with the increasing propensity of HCC apoptosis and elevated DDR. Therefore, these results indicated that 14-3-3ε via its simultaneous associations with some BP/pathway-specific factors to be involved in regulation of gene expression, DNA repair, DNA damaged ends recognition and processing, and cell cycle arrest in response to BLM-induced DNA damage. Meanwhile, these results also demonstrated the accuracy of our proteomic dataset.

Our observations described above established the links between 14-3-3ε and a broad range of BPs or pathways of HCC in response to BLM-induced DNA damage. We then employed STRING tool (http://string.embl.de/) to construct the BLM-induced 14-3-3ε interaction network (Figure 3B). By linking 14-3-3ε to each of the newly validated 14-3-3ε interacting partners (HDACs, NONO, DDB1, TAK1 and TAB1), we found 14-3-3ε is the most interconnected node protein. 14-3-3ε via its associations with HDACs, DDB1, NONO, TAK1 and TAB1 along with other BP/pathway-specific factors is integrated into diverse sub-networks associated with chromosome remodeling (epigenetic control of gene expression), DNA repair, DNA/RNA binding and processing (recognition of DNA damage sites, end processing), signal transduction, protein degradation, cell cycle, and apoptosis involved in the programme of BLM-induced DDR. More importantly, proteins known associated with these key node proteins in several sub-networks (*e.g.,* RBBP4, MTA2, SMARCC2 and PA2G4 with HDAC1[41]-[43], SFPQ and MATR3 with NONO [46], TAB1 and TAB3 with TAK1 [50], [51]) were all identified in BLM-induced 14-3-3ε interactome (Table S3). Interestingly, a key node protein providing the inter-connectivity for 14-3-3ε to other sub-networks was identified as proliferating cell nuclear antigen (PCNA). It is known that PCNA via its associations with many proteins or protein complexes such as PARP1 [52], XRCC5/6 (Ku70/Ku80) [53], [54], MCMs [55] and HDAC1 [56], [57] is involved in regulation of DNA repair, DNA replication and gene expression in response to DNA damage. In our newly constructed network, 14-3-3ε via the node protein DDB1 is interconnected with PCNA-involved multiple processes in response to BLM-induced DDR.

In summary, biological validations and network analysis revealed that 14-3-3ε coordinates the cross-talk among multiple pathway-modules or sub-networks to mediate cell fate decision at BLM-induced crossroad towards either apoptosis or survival with DNA repair.

### 14-3-3ε enhances its binding to the activated form of TAK1 (MAP3K7) through the TAK1's kinase domain in the BLM-stimulated HCC cells

Given that most of the 14-3-3ε-coordinated BPs/pathways identified above are associated with BLM-induced cell fate decisions for either apoptosis or survival. We then explored exactly how 14-3-3ε broadly affects the cellular propensity towards BLM-induced apoptosis. It caught our special attention that 14-3-3ε is well-connected in the sub-networks involved in the signal transduction/amplification process upstream of multiple BPs or pathways for chromosome remodeling, gene regulation, DNA repair, protein degradation, and cell fate decision (Figure 3). Additionally, BLM-induced activation of ERK and JNK (Figure 1B) indicated the involvement of MAPK signaling in the BLM-induced DDR. Also both TAK1 (MAP3K7) and one of its interacting proteins, TAB1 (MAP3K7IP1), showed the BLM-inducible interactions with 14-3-3ε, which were consistent in both MS and immunoblotting analyses (Figure 5A-B and Figure S4F-G). Additionally, we previously observed that TNF-α stimulation enhanced the associations of 14-3-3ε with TAK1 and TAK1's interacting protein PPM1B (phosphatase) in 293T cells [58]. The interactions between 14-3-3ε and TAK1/PPM1B dynamically correlated to the time course-dependent changes in NF-κB activity in response to TNF-α stimulation, suggesting that 14-3-3ε functions as a scaffold protein to recruit phosphatase PPM1B to TAK1, thereby dynamically modulating TAK1-mediated NF-κB activity according to the extent of the cellular response to TNF-α [58]. Furthermore, a recent work showed that TAK1 was activated in response to DNA damage induced by genotoxic agents such as etoposide (VP16) and camptothecin (CPT), and the activation of TAK1 is ATM- and NEMO-dependent [59]. Since BLM is also a similar genotoxic agent, which motivated us to investigate the functional role of the interaction between 14-3-3ε and TAK1 during BLM-induced DDR.

TAK1, a serine/threonine kinase, acts as an essential component of the MAP kinase signal transduction pathway. TAK1 is activated to exert signal transduction in a phosphorylation-dependent manner [60], [61]. In most cases, phosphorylation on specific site of targeted proteins is essential for 14-3-3 binding [11], [62]. Since we observed that TAK1 enhanced its association with 14-3-3ε in response to BLM treatment (Figure 5A), we then tested if BLM could trigger the activation of TAK1 and if activation of TAK1 would be required for its BLM-inducible binding to 14-3-3ε. Firstly, using the level of phospho-TAK1 at Thr187 as an indicator, we examined how TAK1 is activated in the HCC response to BLM stimulation. As shown in Figure 5C, TAK1 displayed a time course-dependent activation along with the activation of its substrates including JNK and IκB (Figure 1B), indicating that TAK1 was induced to be active in the presence of DNA DSBs. Secondly, we examined how the binding of 14-3-3ε to TAK1 is associated with TAK1 activation. FLAG-14-3-3ε expressed HCC cells were either incubated only with medium or treated with BLM. Then, cells were lysed and immunoprecipitated with anti-FLAG beads. As the results shown in Figure 5D, the activity of TAK1 was further increased along with the increasing BLM concentration and incubation time as evidenced by phospho-TAK1-T187 antibody (whole cell lysate, WCL). In the immunoprecipitates pulled-down by anti-FLAG beads, the binding between 14-3-3ε and TAK1 (total TAK1) was enhanced when cells treated with BLM, and this increase was caused by phosphorylation-dependent activation of TAK1. These results showed that the activated TAK1 triggered by BLM promotes its association with 14-3-3ε.

**Figure 6:**
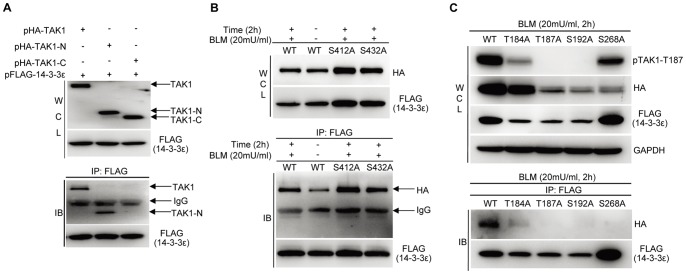
BLM induces site-specific phosphorylations in the kinase domain of TAK1, which in turn mediates the binding of TAK1 to 14-3-3ε. **A.** The kinase domain of TAK1 mediates its binding to 14-3-3ε. **B.** Neither S412 nor S432 located in the C-terminal domain of TAK1 is involved in BLM-induced binding to 14-3-3ε. C. BLM induces the phosphorylations on T184, T187, S192 or S268 residues residing in the kinase domain of TAK1, which mediate its binding to 14-3-3ε.

We then searched for the interacting domains between 14-3-3ε and TAK1. Accordingly, we generated the HA-tagged constructs expressing the full-length TAK1 (TAK1), and the truncated forms of TAK1 including its kinase domain (TAK1-N, 1-300AA) and the C-terminal domain (TAK1-C, 301-579AA), and then co-transfected each one of these constructs respectively with FLAG-tagged 14-3-3ε into HCC cells. As the results shown in Figure 6A, in the immunoprecipitates pulled-down using anti-FLAG (14-3-3ε) beads, both full-length and TAK1-N of TAK1 were detected, indicating TAK1 kinase domain is directly involved in the TAK1 binding to 14-3-3ε.

Phosphorylations at certain Ser/Thr residues of TAK1, including Thr184, Thr187 and Ser192 that reside in the TAK1-N domain (activation loop) [61], [63]–[65] and Ser412 that locates in TAK1-C domain [66], [67], are known essential for the activation of TAK1. Interestingly, Ser412 on TAK1 was identified as a BLM-induced phosphorylation site in our MS analysis (Figure S6 and Table S1). In addition, most of 14-3-3′s interacting partners are the phosphorylated proteins containing characteristic 14-3-3 binding motifs, RXXpS/TXP or RXXXpS/TXP (pS/T represents phospho-Ser/Thr, X stands for any amino acid) [68]. Based on this knowledge, we then sought to determine the potential sites or motifs on TAK1 responsible for mediating its binding to 14-3-3ε in the BLM-treated cells.

We constructed a series of HA-tagged phosphoSer/Thr-depleted TAK1 mutants including T184A, T187A, and S192A in the TAK1-N domain, S268A involved in putative 14-3-3 binding motif, as well as S412A and S432A in the TAK1-C domain, in which each of the serine or threonine residues was substituted by alanine.

Following the co-transfection of either wild-type (WT) TAK1 or each of the mutants respectively with FLAG-14-3-3ε, comparative immunoblotting analyses of the immunoprecipitates pulled-down by anti-FLAG beads from BLM-treated versus non-treated cells revealed the followings: Firstly, although BLM treatment clearly enhanced the bindings between 14-3-3ε and either wild-type (WT) TAK1 or each of the mutants at Ser412 or Ser432, no difference was found in terms of their binding strengths to 14-3-3ε (Figure 6B). These results indicated that the phosphorylations of Ser412 and/or Ser432 residues residing in the C-terminal domain, which were triggered by BLM, are not involved in the binding between 14-3-3ε and TAK1. In contrast, the 14-3-3ε binding of the mutants containing either T184A, or T187A or S192A was significantly weakened or completely abolished compared to that of wild-type (WT) TAK1 (Figure 6C). Meanwhile, the depletion of phosphorylation propensity at either Thr184 or Thr187 or Ser192 clearly impaired or abolished BLM-triggered TAK1 activation as evidenced by phospho-TAK1-T187 antibody (Figure 6C, whole cell lysate, WCL). Interestingly, the S268A mutation affected more the activation of TAK1 with no binding to 14-3-3ε, implying that Ser268 could be a novel BLM-inducible phosphorylation site which may play a coordinative role with the phosphorylated sites resided in activation loop such as Thr184/Thr187/Ser192 to facilitate the TAK1 binding to 14-3-3ε.

**Figure 7:**
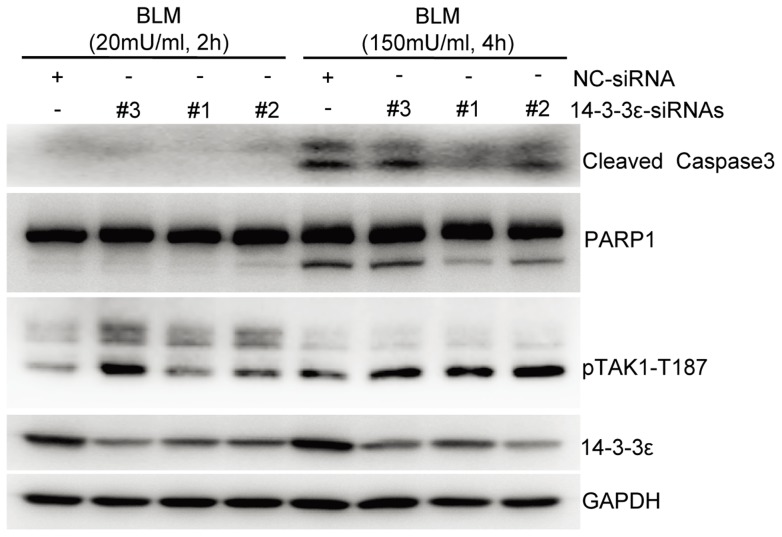
14-3-3ε negatively regulates the anti-apoptotic activity of TAK1 in HCC cells. Either negative-control siRNA (NC) or 14-3-3ε-siRNAs (#1, #2 and #3) was transfected into HCC cells, respectively. 48 hours after transfection, the HCC cells were treated with BLM at indicated dose or time points. Cells were lysed to analyze with corresponding antibodies by immunoblotting. Cellular apoptosis status was indicated by the cleavage of Caspase 3 and its substrate PARP1.

Given that the known role of TAK1 in promoting cell survival (anti-apoptotic activity) when cells were exposed to various stresses and inflammatory cytokines [59], [69], we tested if the removal of the 14-3-3ε binding affects the activation status of TAK1 and its concurrent anti-apoptotic activity in HCC cells. As shown in Figure 7, compared to those in wild-type cells, knocking-down 14-3-3ε by siRNA resulted in an increase of TAK1 activity in the cells exposed to a high-dose BLM at 150 mU/ml for 4 h, leading to a resistance to BLM-induced apoptosis as indicated by both cleavages of Caspase 3 and PARP1. For the first time our results demonstrated that in a phosphorylation-dependent manner 14-3-3ε negatively regulates the TAK1 activity to promote the apoptosis of the HCC cells stimulated by a high-dose BLM. Together with our previous results [58], we concluded that 14-3-3ε may recruit particular phosphatase(s) such as PPM1B to the TAK1 complexes, which rapidly de-phosphorylates TAK1 to promote the BLM-induced apoptosis.

## Discussion

BLM stimulation usually triggers a common DDR programme involving detection of DNA damage, signal amplification/transduction, and concurrent pathways for DNA repair, chromatin remodeling, cell fate decision, *etc*. Based on our proteomic identification of the key composition of the BLM-induced 14-3-3ε interactome, the data-dependent functional categorization and interaction mapping analysis have revealed that 14-3-3ε indeed plays both central and coordinated roles in a network involving multiple pathways or BPs for signal transduction, chromosome remodeling, DNA/RNA binding and processing, DNA repair, cell cycle, protein ubiquitination/degradation, cytoskeleton and cell motility, and apoptosis (Figure 3, Table S3). Using biological assays including site-directed mutagenesis, siRNA, co-immunoprecipitation, and immunoblotting (Figure 4–7), we have validated the physiologically relevant accuracy of our quantitative proteomics approach in dissecting a dynamic interactome of 14-3-3ε.

**Figure 8:**
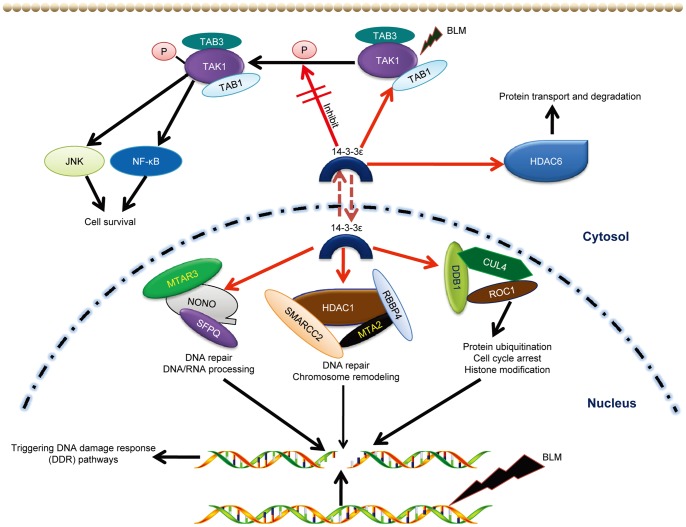
Mechanistic model underlying how 14-3-3ε integrates multiple biological processes (BPs) to coordinate BLM-induced DDR. The red arrow from 14-3-3ε towards specific proteins indicates our validated BLM-induced 14-3-3ε interactors. The components including SMARCC2/ MTA2/RBBP4[41]-[43], MTAR3/SFPQ [46], and TAB3 (MAP3K7IP3) [51] that were previously reported to form complexes with HDAC1, NONO and TAK1 respectively were also indentified by MS.

On a dynamic systems view, the data-dependent construction of the 14-3-3ε interacting network allows us to postulate a mechanistic model as illustrated in Figure 8, where 14-3-3ε coordinates/integrates different pathway modules/sub-networks in the DDR of HCC cells to BLM stimulation.

First, to transduct the signal of detection of BLM-induced DNA damage, multiple MAPK pathways are activated where TAK1, a member of MAP3K family was identified in the BLM-induced 14-3-3ε interactome. The “zoom-in” investigation of the function of the interaction between 14-3-3ε and TAK1 revealed that 14-3-3ε negatively regulates the kinase activity of TAK1 through its BLM-induced binding to the kinase domain of TAK1. Given the known role of TAK1 in promoting the survival of the cells exposed to various stresses and inflammatory cytokines [59], [70], we conclude that the interaction between 14-3-3ε and TAK1 enhanced by a high-dose BLM plays a critical role in inducing apoptosis of the BLM-exposed HCC cells (Figure 7).

Further, extending from the upstream phosphorylation-dependent 14-3-3ε interactions with key MAPK modules for signal transduction, 14-3-3ε also provides the links to key node proteins (HDAC1/HDAC6, NONO, and DDB1) in the cell fate-determining sub-networks associated with the BPs of chromosome remodeling, DNA/RNA binding and processing, DNA repair, and protein degradation. Notably, our identification of the BLM-induced interactions of 14-3-3ε with nuclear localized proteins including HDAC1 and NONO [71], [72] indicated that 14-3-3ε may translocate into nucleus to mediate the activity/function of HDAC1/NONO in the BPs of chromosome remodeling and DNA repair for cell fate decisions. Although 14-3-3 family proteins are known primarily localized in the cytoplasm, our observations of 14-3-3ε interactions with nuclear proteins during the DDR to BLM are in line with a previous report that 14-3-3 could transit in and out of nucleus [73].

In biological validation of the network-linked functions of 14-3-3ε interactions, we have further demonstrated that 14-3-3ε enhances its interactions with these pathway-specific/representing factors including HDAC1, NONO, and DDB1 in HCC DDR to high-dose BLM-induced apoptosis (Figure 4C), implicating that through a specific protein-protein interaction network 14-3-3ε coordinately and dynamically modulates the activity of multiple pathways associated with DNA damage recognition, apoptosis, DSB repair, cell cycle arrest and chromatin remodeling at the BLM-induced crossroad towards either apoptosis or survival/DNA repair.

Strategically, as a discovery-to-hypothesis approach, our phenotype-specific interactome screening provides a systemic basis for “zoom-in” functional characterization of any novel proteins/enzyme interactions in the context of their cooperative and concerted actions within the 14-3-3ε interactome, which in turn facilitates understanding of phenotype-specific systemic function of the interactome on a pathway scale. As a result of our ‘pathway-scale’ characterization of the composition of the BLM-induced 14-3-3ε interactome, we have obtained a data-derived mechanistic model (Figure 8) that provides a systems insight into exactly how at each level of the DDR programme from signal transduction/amplification of DNA damage detection to DNA repair and cell fate determination, 14-3-3ε not only directly participates in but also plays a coordinative role in modulating the activity of these pathways/BPs via its BLM-induced interactions with those pathway-specific regulators. Therefore, 14-3-3ε broadly interfaces and regulates these pathways/BPs in the BLM-triggered DDR of HCC cells. Our systems dataset and the mechanistic model derived reveal not only individual proteins but also those critical links in the network that could be the potential targets for BLM-mediated therapeutic intervention of carcinogenesis.

## Materials and Methods

### Chemicals and reagents

Deuterium-labeled leucine (5,5,5-d_3_) was purchased from Cambridge Isotope Laboratories (Andover, MA); Sequencing-grade trypsin was purchased from Promega (Madison, WI). Fetal bovine serum (A15-151) was purchased from PAA laboratories (PAA Laboratories GmbH, Linz, Austria); Dialyzed FBS (26400-044), and lipofectamine 2000 (11668-019) were obtained from Invitrogen; G418 sulfate (345810) was purchased from Calbiochem; FLAG peptide (F3290) was purchased from Sigma-Aldrich; Bleomycin Sulfate (1076308) was purchased from United States Pharmacopeia (USP).

FLAG antibody (200471) was purchased from Stratagene; anti-FLAG beads (A2220) was obtained from Sigma-Aldrich; 14-3-3ζ antibody (sc-1019) was purchased from Santa Cruz Biotechnology, Inc.; antibodies against 14-3-3 epsilon (ab40117), HDAC1(ab11966) and gamma-H2AX(ab22551) were obtained from Abcam; antibodies against NONO (11058-1-AP), DDB1(11380-1-AP), NCL (10556-1-AP), NFKBIA(IκB-α) (10268-1-AP) and p65(10745-1-AP) were purchased from Proteintech group, Inc.; TAK1(#4505), phospho-Chk1-S345(#2348), phospho-TAK1-T187(#4536), phospho-SPAK/JNK(Thr183/Tyr185) (#9251), phospho-ERK1/2(Thr202/Tyr204) (#9101), Caspase-3(#9662), cleaved Caspase-3 (#9661), and PARP1(#9542) antibodies were purchased from Cell Signaling Tech.; HDAC6 antibody (AP1106a) were purchased from Abgent.

### Plasmid construction

Primers for constructing all expressing plasmids are listed in Table S4.

### Cell culture, selection of stable HCC cell line, BLM treatment, and protein extraction

Hepatocellular carcinoma (HCC) cell line QGY-7703, derived from primary hepatocellular carcinoma of a 35 years old female, was purchased from the Cell Bank of Chinese Academy of Science (Shanghai, China) [74] and grown in RPMI 1640 medium supplemented with 10% FBS. The HCC cell line stably expressing FLAG-14-3-3ε (stable HCC line) was chosen by selecting transfected cells using 400 μg/ml G418.

Stable HCC cells were treated either with or without BLM at indicated dose or for different time periods, the whole cell lysates (WCL) was extracted by lysis buffer (Beyotime P0013) supplemented with protease inhibitor cocktail (Sigma, P8340) and phosphatase inhibitor cocktail (Sigma, P5726).

To detect the nuclear translocation of p65 (NF-κB) in the HCC cells expressing FLAG-14-3-3ε with or without BLM treatment at indicated dose and time points, nuclear fractions were isolated by a nuclear isolation kit (Beyotime, P0027) according to the manufacturer's protocol.

For the isolation of histone/gamma-H2AX (γ-H2AX), cells were lysed by the lysis buffer (PBS containing 0.5% Triton-X100, phosphatase inhibitor cocktail (Sigma, P5726) and 2 mM PSMF) on ice for 15 min. After centrifugation, wash the pellets twice with the lysis buffer. Added 0.2 M HCl into pellets and kept them at 4^o^C overnight to extract histone/γ-H2AX. After extraction, neutralize HCl with NaOH before the following use.

### Stable isotope labeling (AACT), and isolation/purification of BLM-induced 14-3-3ε interacting complexes

The detailed procedure of Leu-d_3_ isotope labeling has been described previously [30], [37], [75]. Briefly, appropriately 1×10^9^ HCC cells stably expressing FLAG-14-3-3ε grown in regular (unlabeled) RPMI-1640 medium were treated with 20mU/ml BLM for 2 h while the untreated cells were maintained in the medium containing Leu-d_3_. Each cell pool was lysed with the lysis buffer (Beyotime, P0013) supplemented with protease inhibitor cocktail (Sigma, P8340) and phosphatase inhibitor cocktail (Sigma, P5726), respectively. Equal amounts of protein derived from each cell pool were incubated with 350 μl anti-FLAG beads (Sigma, A2220) at 4°C for 4 h to immunoprecipitate FLAG-14-3-3ε interacting proteins, respectively. After immunoprecipitation, anti-FLAG beads were washed four times with TBS buffer (50 mM Tris-HCl, pH 7.4, 150 mM NaCl) to eliminate the non-specific binding. Immunoprecipitates were eluted by 100 ug/ml of FLAG peptide, then elutes were combined and concentrated to the appropriate volume for the following SDS-PAGE separation. The procedure of visible bands cutting, in-gel Trypsin digestion, and peptides extraction was performed as previously described [76].

### LC-MS/MS analysis

LC-MS/MS experiments were performed on a hybrid linear quadruple ion trap/Orbitrap (LTQ-Orbitrap) mass spectrometer (Thermo Scientific) coupled to a Shimadzu LC-20AD LC system (Shimadzu, Japan) and SIL-20AC autosampler (Shimadzu, Japan). Peptide mixtures were separated by a PICOFRIT C18 reverse-phase column (0.075×100 mm, New Objective Inc., Woburn, MA) at a flow rate of 300 nL/min with a 110 min-gradient. Samples were loaded in solvent A (95% H_2_O, 5% ACN, 0.1% formic acid), and peptides were eluted by 5% solvent B (5% H_2_O, 95% ACN, 0.1% formic acid) for 5 min followed by a linear gradient to 45% solvent B in the next 90 min, ramping to 95% solvent B in 4 min and dropping to 90% solvent B for 4 min before re-equilibrating the system to 10% solvent B for 7 min. The entire eluant was sprayed into the mass spectrometer via a dynamic nanospray probe (Thermo Fisher Scientific) and analyzed in positive mode. The 3 most abundant precursor ions detected in the full MS survey scan (m/z range of 400-2000, R = 100 000) were isolated for further MS/MS analyzing. Spectra were acquired under automatic gain control (AGC) for survey spectra (AGC: 10^6^) and for MS/MS spectra (AGC: 10^4^). The spray voltage was 1.6 kV and the temperature of ion transfer capillary was 180°C.

### Database search and protein identification

Tandem mass spectra were extracted by Bioworks (version 3.3.1 SP1, Thermo Scientific) and submitted to human International Protein Index database (IPI human 3.45, 71983 entries) using TurboSequest V.28 (rev. 13) search engine. Searches were performed with the following parameters: Trypsin enzyme specificity with allowing 2 missed cleavages; a precursor tolerance of 10 ppm and fragment tolerance of 1 Da; variable modifications include Leu-d_3_ (+3.0188), phosphorylated Ser/Thr/Tyr (+79.9663) and oxidized Met (15.9994). TurboSequest results were filtered by Xcorr, peptide probability and Delta Cn. Xcorr was set to 1.9, 2.6, 3.6 for 1+, 2+ and 3+ charged precursor ions, respectively; peptide probability<0.001, delta Cn >0.1, and keratin (most of them are contaminants) was excluded. The data's false positive rate was estimated under 1% by reverse database search, the reverse database was generated from IPI human 3.45 (71983 entries). Each protein identified requires the match of at least two unique peptides.

### Protein quantification

The light-to-heavy ratio (L/H) of each leucine-containing peptide was calculated using the extracted ion chromatogram (XIC) of both versions with PepQuan SILAC software integrated in Bioworks 3.3.1 SP1. For the default parameter, we selected leucine as the “modified” amino acid residue with a mass shift of 3.0188 Da between its light and heavy isotope version with the mass tolerance of 0.01Da. Protein ratio (L/H) was calculated by averaging the ratios of all leucine-containing peptides sequenced for each protein, and standard deviation (SD) was given to evaluate the accuracy of protein ratio. The proteins with their L/H SD larger than 20% were further validated by manually checking their MS signals. In addition, to assess the quality of our quantitative results, we re-processed all the raw data generated by LTQ-Orbitrap through the Proteome Discoverer platform (version 1.3, Thermo Scientific), which integrates both Mascot and Sequest algorithms. Therefore, the output from different search engines can be cross-validated or merged to and maximize the protein identifications. The “Quantitation” node in Proteome Discoverer was used for global protein quantitation. Only unique peptides are used for protein ratio calculation. And the protein ratio is the median value of peptide ratios. The protein ratio variability is calculated as a coefficient-of-variation (CV) for log-normal distributed data. The quantitative results from Proteome Discoverer can be found in Figure S3.

### Functional categorization and network analysis

The all identified BLM-induced HCC-specific 14-3-3ε inteactors were submitted to Gene Ontology (http://www.geneontology.org/) and DAVID (http://www.david.abcc.ncifcrf.gov) for categorization according to their previously known associations with different functions. Network analysis on our proteomic dataset by using STRING mapping tool (http://string.embl.de/), the scheme of BLM-induced 14-3-3ε interacting network was constructed by the Cytoscape 2.6 (http://www.cytoscape.org/).

### Cell transfection and RNA interference

Co-transfection was carried out in 60 mm dishes. Briefly, 4.0 μg HA-tagged constructs were co-transfected with 4.0 μg FLAG-tagged 14-3-3ε into QGY-7703 cells using Lipofectamine 2000 transfection reagent according to the manufacture's protocol, respectively. Cells were analyzed after 48 h transfection.

The small interfering RNAs (siRNAs) specific to human 14-3-3ε were designed and synthesized by Shanghai GenePharma Co., Ltd, China. The siRNAs target sequences were as follows: #1(5′-GCUUAGGUCUUGCUCUCAATT-3′, 5′-UUGAGAGCAAGACCUAAGCTT-3′), #2(5′-GGAGGAGAAGACAAGCUAATT-3′, 5′-UUAGCUUGUCUUCUCCUCCTT-3′) and #3(5′-CGCUGAGUGAAGAAAGCUATT-3′/ 5′-UAGCUUUCUUCACUCAGCGTT-3′). Negative Control (NC) siRNA (AM4611) was purchased from Ambion, Inc. The siRNAs (40nM) were transfected into QGY-7703 cells using Lipofectamine 2000 transfection reagent. Cells were analyzed after 48 h transfection.

### Co-immunoprecipitation and Immunoblotting

The procedures of protein isolation and immunoprecipitation were essentially the same as described in the purification of FLAG-14-3-3ε complexes for MS identification. The immunoprecipitated FLAG-14-3-3ε complexes were eluted by FLAG peptide or by directly boiling the anti-FLAG beads. Samples were separated by SDS-PAGE and transferred on PVDF membrane (Immobilon-P^SQ^ Membrane, 0.2 µm). The membranes were blocked with 5% nonfat milk and probed with specified primary antibody followed by incubation with secondary antibody conjugated with horseradish peroxidase, and then ECL substrate was added on membrane and exposed by ECL system.

## Supporting Information

Figure S1 **FLAG tag fused on the N-terminus of 14-3-3ε has no effect on the function of 14-3-3ε.** **A.** The expression level of exogenous 14-3-3ε (FLAG-14-3-3ε) is less than that of endogenous form in HCC cells. Due to FLAG tag is immediately fused on the N-terminus of 14-3-3ε and the short length nature of FLAG tag, the exogenous (FLAG-14-3-3ε) and endogenous 14-3-3ε were not able to be separated by conventional SDS-PAGE gel. To estimate the expression level of FLAG-14-3-3ε, we roughly normalized the densitometry of loading control (GAPDH) between parental cells and FLAG-14-3-3ε expressing cells according to immunoblotting (IB) assay as 1.0, and then, we determined that the expression level of FLAG-tagged 14-3-3ε is about 50 percent of that of endogenous 14-3-3ε. **B.** FLAG-14-3-3ε heterodimerized with endogenous 14-3-3ζ in FLAG-14-3-3ε expressing HCC.
(PDF)

Figure S2 **Schematic design of dual-tagging quantitative proteomic approach.** HCC cells stably expressing FLAG-14-3-3ε maintained in “light” (Leu) medium were treated with 20mU/ml BLM for 2h. In parallel, cells maintained in “heavy” medium (Leu-d_3_) were left untreated. The FLAG-14-3-3ε complexes were immunoprecipitated (IP) from the whole cell lysate derived from each cell pool using anti-FLAG beads and eluted by 1×FLAG peptide, respectively. IP products were mixed at 1:1 based on the total protein mass followed by SDS-PAGE separation, in-gel trypsin digestion, and LC-MS/MS analysis.
(PDF)

Figure S3 **The plot of AACT/SILAC ratio distribution of identified peptides/proteins and the ratio variability of all quantifiable proteins.** All the raw data generated by LTQ-Orbitrap (Thermo Scientific) was re-processed on Proteome Discoverer platform (v1.3, Thermo Scientific) and searched against the UniProt protein database (Released in 2010_9, 20286 human sequences). The top 32 most abundant proteins (PSM≥100 and unique peptide≥2) were highlighted by red diamonds. Those proteins with significant changes identified by both platforms (Bioworks v3.3.1SP1 and Proteome Discoverer v1.3) were indicated. As a result, over 94% of overall quantifiable proteins are showing less that 40% ratio variability, and more than 70% have variabilities less than 20%. Also the most abundant proteins in the complex have ratio varabilities varying from 3.2% to 21.9%. The average is at 13.39 (±4.88 SD). This variation is consistent with the Bioworks result and our manual inspection. Therefore, we chose the cutoff value at three times of the variability or 1.4.
(PDF)

Figure S4 **Representative MS spectra of BLM-induced 14-3-3ε interactors.** **A.** 14-3-3ε (bait protein). **B.** HDAC1. **C.** NONO. **D.** HDAC6. **E.** DDB1. **F.** MAP3K7 (TAK1). **G.** MAP3K7IP1 (TAB1).
(PDF)

Figure S5 **MS and MS/MS spectra of the peptides matched to HDAC1.** All raw data generated on LTQ-Orbitrap (Thermo Scientific) were re-processed using Proteome Discoverer software (v1.3, Thermo Scientific), which resulted in one more unique peptide (YYAVNYPLR) of HDAC1 compared to the previous results from Bioworks (v3.3.1SP1) search (Figure S4B). The AACT/SILAC pairs of both peptides and a representative MS/MS are shown.
(PDF)

Figure S6 **Ser412 on TAK1 was identified as a BLM-induced phosphorylation site by MS/MS.** The MS/MS spectra of both phosphorylated and unphosphorylated sequence (SIQDLTVTGTEPGQVSSR) were shown.
(PDF)

Table S1 **The list of all proteins identified in 14-3-3ε immunoprecipitates by MS.**
(XLSX)

Table S2 **Analysis and categorization of all quantified proteins identified in 14-3-3ε immunoprecipitates.**
(XLSX)

Table S3 **Categorization of the identified BLM-induced 14-3-3ε interactors and their associations with different biological processes or pathways.** All of MS-indentified BLM-induced HCC-specific 14-3-3ε interactors were grouped into the specific biological processes (BPs) or pathways by using online database-Gene Ontology (http://geneontology.org/) and DAVID (http://david.abcc.ncifcrf.gov).
(PDF)

Table S4 **The list of TAK1 sequence and primers for constructing the expressing plasmids.**
(PDF)
