# Supplementary material for: 14-3-3ε Mediates the Cell Fate Decision-Making Pathways in Response of Hepatocellular Carcinoma to Bleomycin-Induced DNA Damage
Source: PLoS One. 2013 Mar 5;8(3):e55268. doi: 10.1371/journal.pone.0055268 (PMC3589417; doi:10.1371/journal.pone.0055268)
Supplement: Figure S1 — FLAG tag fused on the N-terminus of 14-3-3ε has no effect on the function of 14-3-3ε. A. The expression level of exogenous 14-3-3ε (FLAG-14-3-3ε) is less than that of endogenous form in HCC cells. Due to FLAG tag is immediately fused on the N-terminus of 14-3-3ε and the short length nature of FLAG tag, the exogenous (FLAG-14-3-3ε) and endogenous 14-3-3ε were not able to be separated by conventional SDS-PAGE gel. To estimate the expression level of FLAG-14-3-3ε, we roughly normalized the densitometry of loading control (GAPDH) between parental cells and FLAG-14-3-3ε expressing cells according to immunoblotting (IB) assay as 1.0, and then, we determined that the expression level of FLAG-tagged 14-3-3ε is about 50 percent of that of endogenous 14-3-3ε. B. FLAG-14-3-3ε heterodimerized with endogenous 14-3-3ζ in FLAG-14-3-3ε expressing HCC. (PDF) [file pone.0055268.s001.pdf]

Figure S1

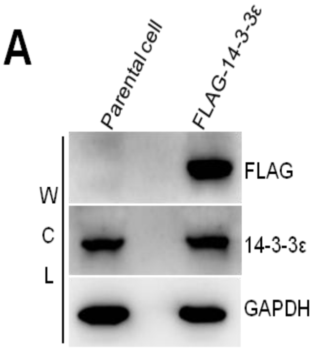

protein	Ratio (FLAG-14-3-3ε/Parental)	Ratio normalized by GAPDH
GAPDH	0.86	1.00
14-3-3ε	1.23	1.43

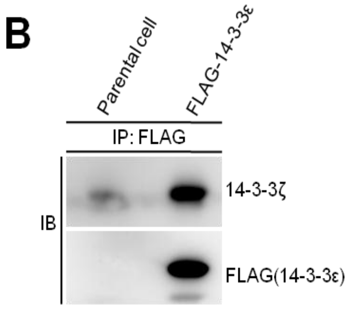

Figure S1. FLAG tag fused on the N-terminus of 14-3-3ε has no effect on the function of 14-3-3ε. A. The expression level of exogenous 14-3-3ε (FLAG-14-3-3ε) is less than that of endogenous form in HCC cells. Due to FLAG tag is immediately fused on the N-terminus of 14-3-3ε and the short length nature of FLAG tag, the exogenous (FLAG-14-3-3ε) and endogenous 14-3-3ε were not able to be separated by conventional SDS-PAGE gel. To estimate the expression level of FLAG-14-3-3ε, we roughly normalized the densitometry of loading control (GAPDH) between parental cells and FLAG-14-3-3ε expressing cells according to immunoblotting (IB) assay as 1.0, and then, we determined that the expression level of FLAG-tagged 14-3-3ε is about 50 percent of that of endogenous 14-3-3ε. **B.** FLAG-14-3-3ε heterodimerized with endogenous 14-3-3ζ in FLAG-14-3-3ε expressing HCC.
