# Supplementary material for: 14-3-3ε Mediates the Cell Fate Decision-Making Pathways in Response of Hepatocellular Carcinoma to Bleomycin-Induced DNA Damage
Source: PLoS One. 2013 Mar 5;8(3):e55268. doi: 10.1371/journal.pone.0055268 (PMC3589417; doi:10.1371/journal.pone.0055268)
Supplement: Figure S2 — Schematic design of dual-tagging quantitative proteomic approach. HCC cells stably expressing FLAG-14-3-3ε maintained in “light” (Leu) medium were treated with 20mU/ml BLM for 2h. In parallel, cells maintained in “heavy” medium (Leu-d3) were left untreated. The FLAG-14-3-3ε complexes were immunoprecipitated (IP) from the whole cell lysate derived from each cell pool using anti-FLAG beads and eluted by 1×FLAG peptide, respectively. IP products were mixed at 1:1 based on the total protein mass followed by SDS-PAGE separation, in-gel trypsin digestion, and LC-MS/MS analysis. (PDF) [file pone.0055268.s002.pdf]

Figure S2

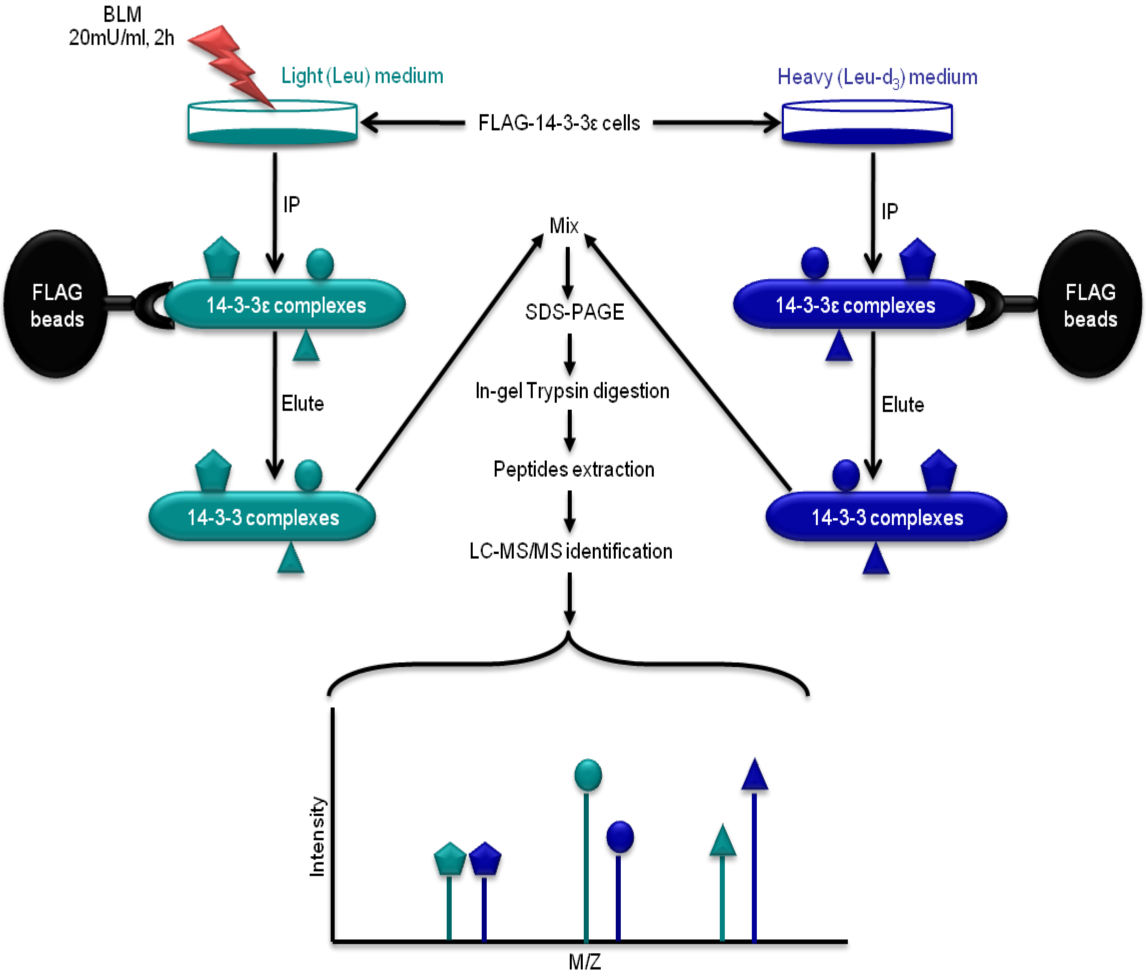

Figure S2. Schematic design of dual-tagging quantitative proteomic approach. HCC cells stably expressing FLAG-14-3-3ε maintained in "light" medium (Leu) were treated with 20mU/ml BLM for 2h. In parallel, cells maintained in "heavy" medium (Leu-d_3) were left untreated. The FLAG-14-3-3ε complex were immunoprecipitated (IP) from the whole cell lysate derived from each cell pool using anti-FLAG beads and eluted by 1×FLAG peptide, respectively. IP products were then mixed at 1:1 based on the total protein mass followed by SDS-PAGE separation, in-gel trypsin digestion, and LC-MS/MS analysis.
