# Supplementary material for: 14-3-3ε Mediates the Cell Fate Decision-Making Pathways in Response of Hepatocellular Carcinoma to Bleomycin-Induced DNA Damage
Source: PLoS One. 2013 Mar 5;8(3):e55268. doi: 10.1371/journal.pone.0055268 (PMC3589417; doi:10.1371/journal.pone.0055268)
Supplement: Figure S3 — The plot of AACT/SILAC ratio distribution of identified peptides/proteins and the ratio variability of all quantifiable proteins. All the raw data generated by LTQ-Orbitrap (Thermo Scientific) was re-processed on Proteome Discoverer platform (v1.3, Thermo Scientific) and searched against the UniProt protein database (Released in 2010_9, 20286 human sequences). The top 32 most abundant proteins (PSM≥100 and unique peptide≥2) were highlighted by red diamonds. Those proteins with significant changes identified by both platforms (Bioworks v3.3.1SP1 and Proteome Discoverer v1.3) were indicated. As a result, over 94% of overall quantifiable proteins are showing less that 40% ratio variability, and more than 70% have variabilities less than 20%. Also the most abundant proteins in the complex have ratio varabilities varying from 3.2% to 21.9%. The average is at 13.39 (±4.88 SD). This variation is consistent with the Bioworks result and our manual inspection. Therefore, we chose the cutoff value at three times of the variability or 1.4. (PDF) [file pone.0055268.s003.pdf]

Figure S3

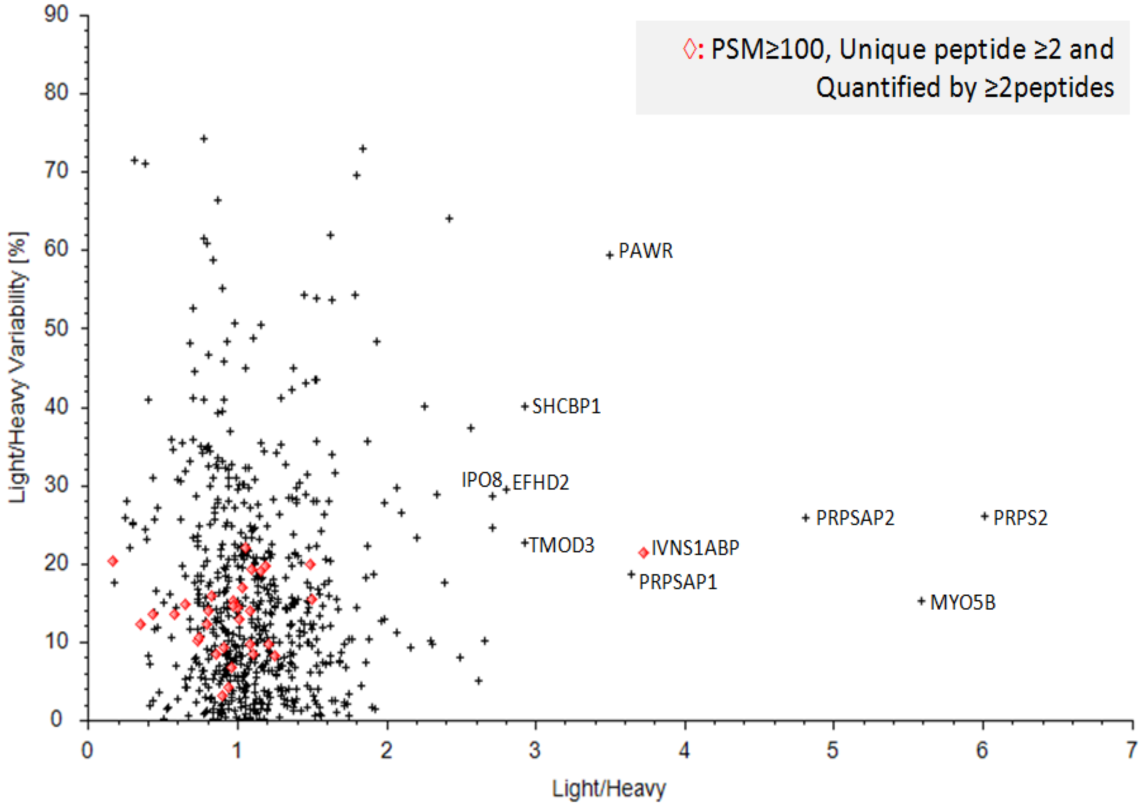

Figure S3. The plot of AACT/SILAC ratio distribution of identified peptides/proteins and the ratio variability of all quantifiable proteins. All the raw data generated by LTQ-Orbitrap (Thermo Scientific) was re-processed on Proteome Discoverer platform (v1.3, Thermo Scientific) and searched against the UniProt protein database (Released in 2010_9, 20286 human sequences). The top 32 most abundant proteins (PSM≥100 and unique peptide≥2) were highlighted by red diamonds. Those proteins with significant changes identified by both platforms (Bioworks v3.3.1SP1 and Proteome Discoverer v1.3) were indicated. As a result, over 94% of overall quantifiable proteins are showing less that 40% ratio variability, and more than 70% have variabilities less than 20%. Also the most abundant proteins in the complex have ratio varabilities varying from 3.2% to 21.9%. The average is at 13.39 (±4.88 SD). This variation is consistent with the Bioworks result and our manual inspection. Therefore, we chose the cutoff value at three times of the variability or 1.4.
