# Supplementary material for: 14-3-3ε Mediates the Cell Fate Decision-Making Pathways in Response of Hepatocellular Carcinoma to Bleomycin-Induced DNA Damage
Source: PLoS One. 2013 Mar 5;8(3):e55268. doi: 10.1371/journal.pone.0055268 (PMC3589417; doi:10.1371/journal.pone.0055268)
Supplement: Figure S4 — Representative MS spectra of BLM-induced 14-3-3ε interactors. A. 14-3-3ε (bait protein). B. HDAC1. C. NONO. D. HDAC6. E. DDB1. F. MAP3K7 (TAK1). G. MAP3K7IP1 (TAB1). (PDF) [file pone.0055268.s004.pdf]

Figure S4

A

IPI:IPI00000816.1 Gene Symbol=YWHAE, 14-3-3 protein epsilon

K.EALQDVEDENQ.   Charge: 2+

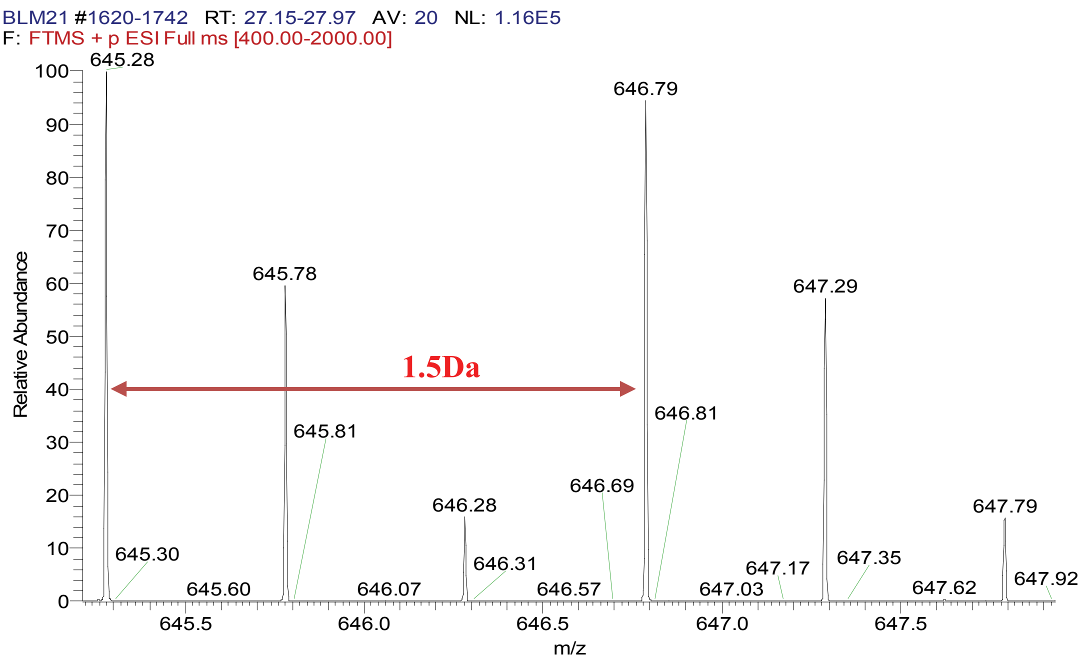

B

IPI:IPI00013774.1 Gene Symbol=HDAC1, Histone deacetylase 1

K.LHISPSNMTNQNTNEYLEK.I   Charge: 3+

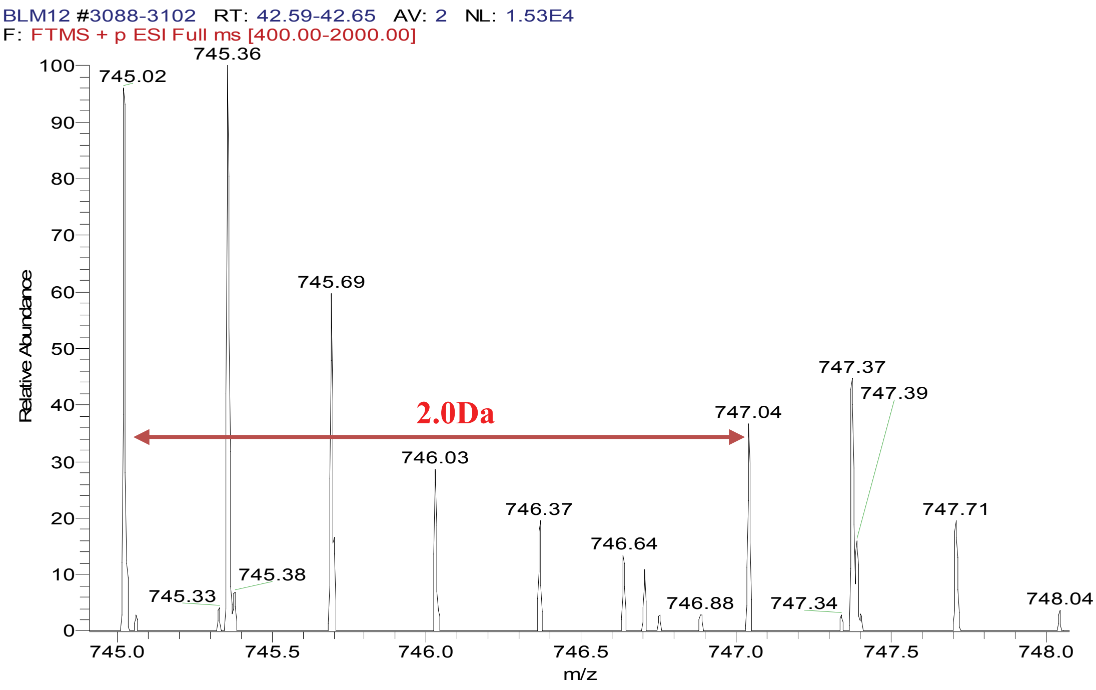

C

IPI:IPI00304596.3 Gene Symbol=NONO, Non-POU domain-containing octamer-binding protein

R.LFVGNLPPDITEEEMR.K Charge: 2+

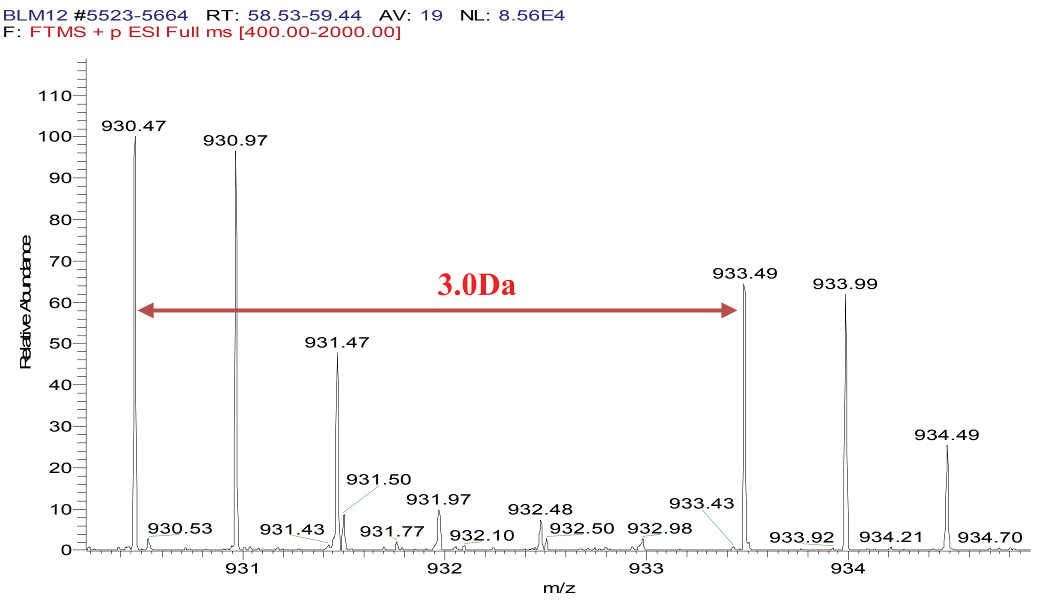

D

IPI:IPI00005711.2 Gene Symbol=HDAC6, Histone deacetylase 6

K.EQLIQEGLLDR.C Charge: 2+

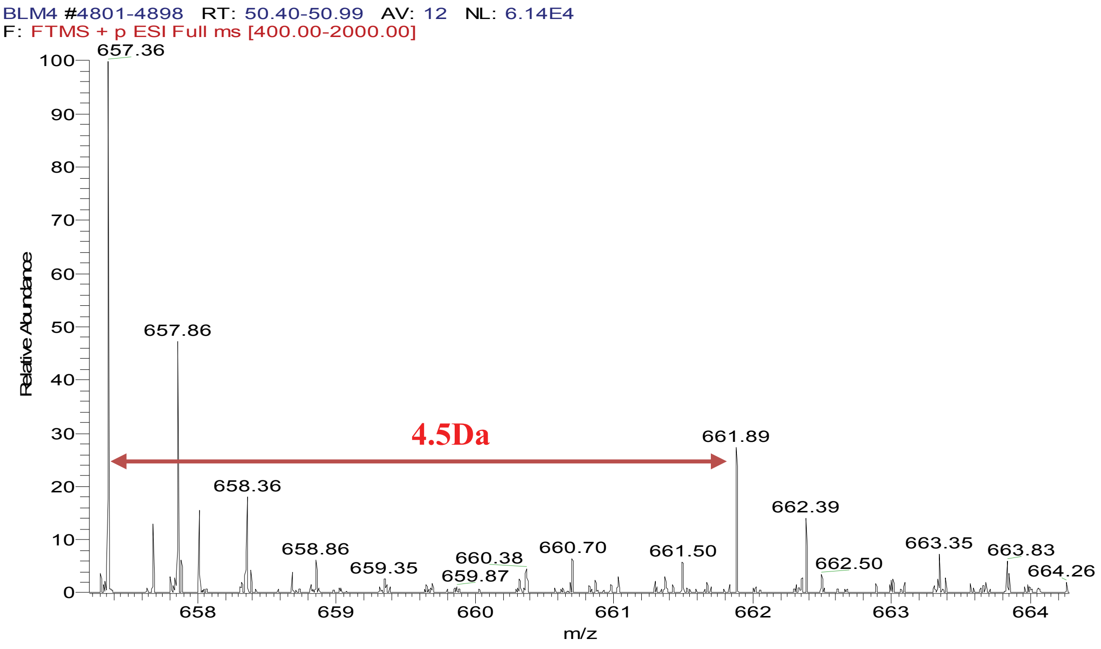

E

IPI:IPI00293464.5 Gene Symbol=DDB1, DNA damage-binding protein 1
K.MQEVVANLQYDDGSGMK.R   Charge: 2+

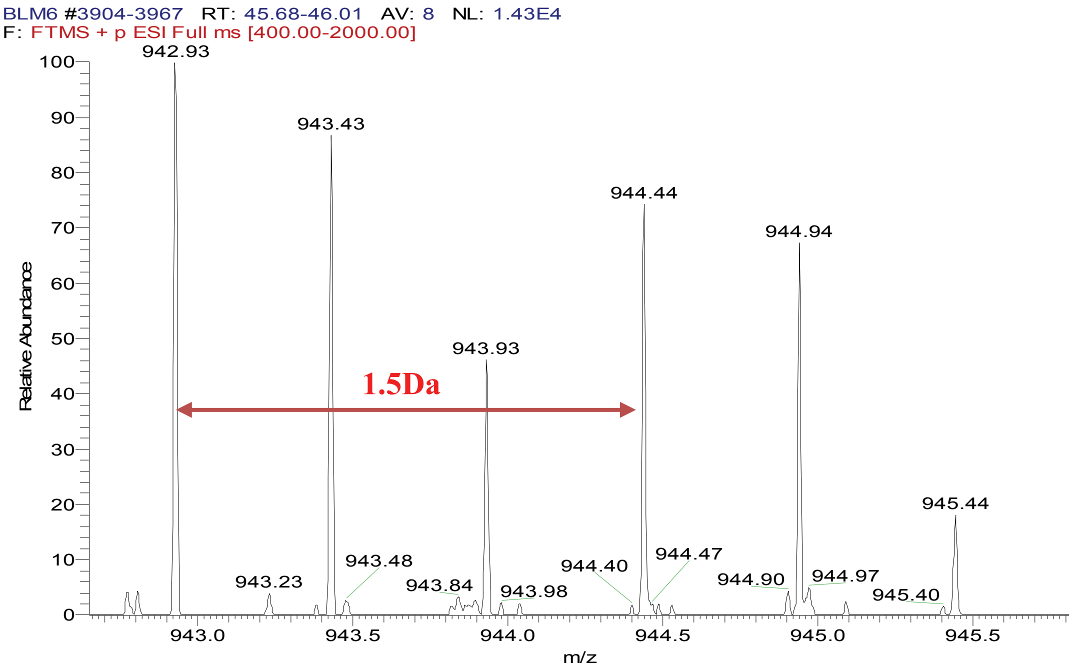

F

IPI:IPI00218566.1 Gene Symbol=MAP3K7, Isoform 1A of Mitogen-activated protein kinase kinase kinase 7
R.SIQDLTVTGTEPGQVSSR.S   Charge: 2+

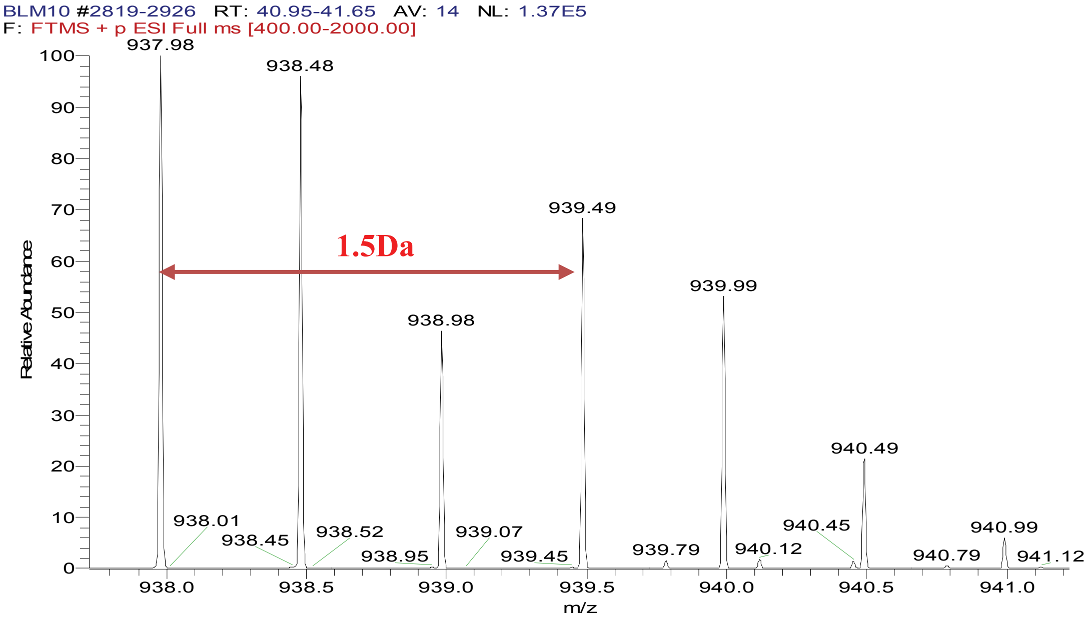

G

IPI:IPI00019459.1 Gene Symbol=MAP3K7IP1, Mitogen-activated protein kinase kinase kinase 7-interacting protein 1

K.QTSLDAVAQAVVDR.V   Charge: 2+

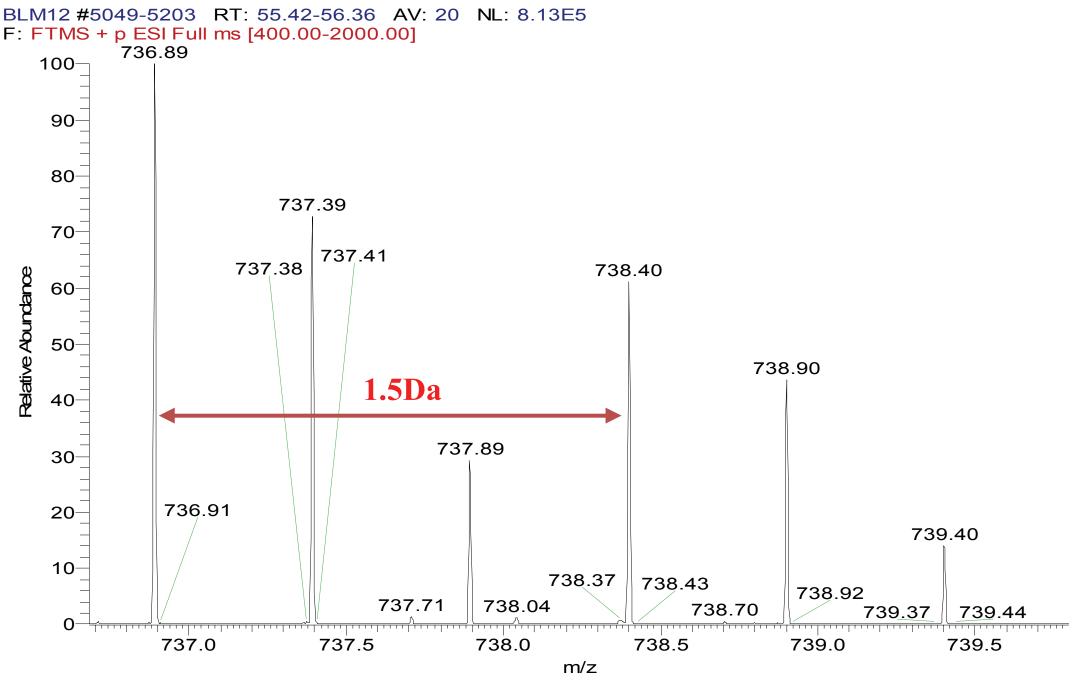

Figure S4. Representative MS spectra of BLM-induced 14-3-3ε interactors. A. 14-3-3ε (bait protein). **B.** HDAC1. **C.** NONO. **D.** HDAC6. **E.** DDB1. **F.** MAP3K7 (TAK1). **G.** MAP3K7IP1 (TAB1).
