# Supplementary material for: 14-3-3ε Mediates the Cell Fate Decision-Making Pathways in Response of Hepatocellular Carcinoma to Bleomycin-Induced DNA Damage
Source: PLoS One. 2013 Mar 5;8(3):e55268. doi: 10.1371/journal.pone.0055268 (PMC3589417; doi:10.1371/journal.pone.0055268)
Supplement: Figure S5 — MS and MS/MS spectra of the peptides matched to HDAC1. All raw data generated on LTQ-Orbitrap (Thermo Scientific) were re-processed using Proteome Discoverer software (v1.3, Thermo Scientific), which resulted in one more unique peptide (YYAVNYPLR) of HDAC1 compared to the previous results from Bioworks (v3.3.1SP1) search (Figure S4B). The AACT/SILAC pairs of both peptides and a representative MS/MS are shown. (PDF) [file pone.0055268.s005.pdf]

Figure S5

Accession	Description	Coverage	# Unique Peptides	# PSMs	L/H	L/H Count	L/H Variability [%]	MW [kDa]	calc. pI
Q13547	Histone deacetylase 1	5.81 %	2	5	1.565	2	0.5	55.1	5.48

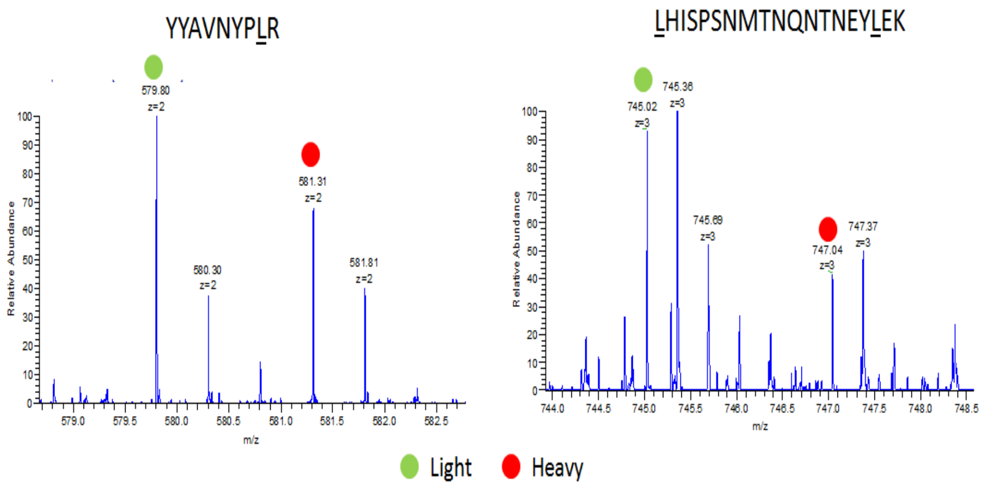

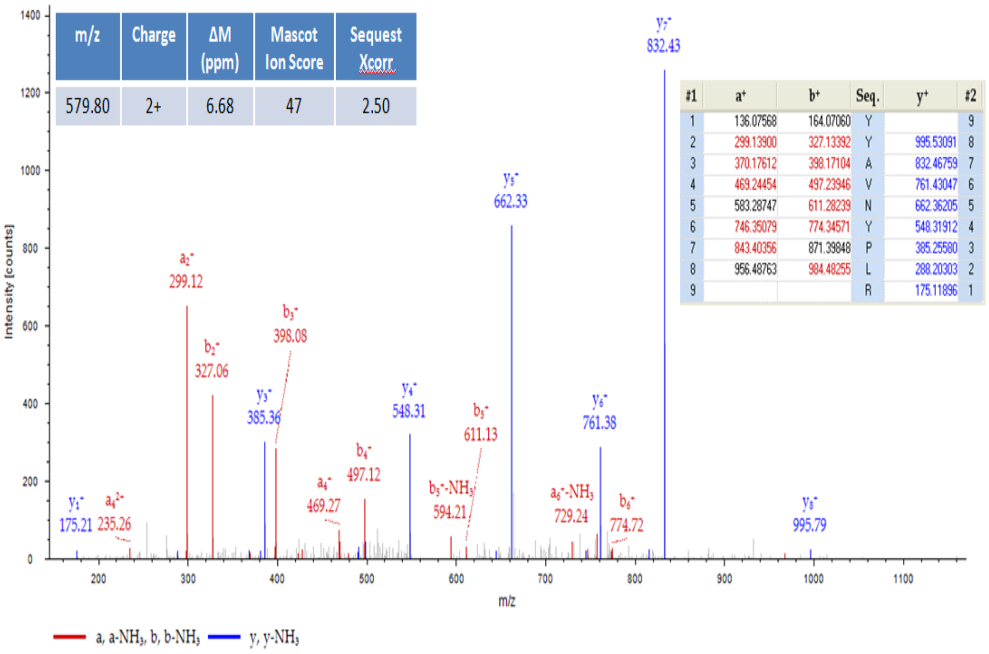

m/z	Charge	ΔM (ppm)	Mascot Ion Score	Sequest Xcorr
579.80	2+	6.68	47	2.50

#1	a+	b+	Seq.	y+	#2
1	136.07568	164.07060	Y		9
2	299.13900	327.13392	Y	995.53091	8
3	370.17612	398.17104	A	832.46759	7
4	469.24454	497.23946	V	761.43047	6
5	583.28747	611.28239	N	662.36205	5
6	746.35079	774.34571	Y	548.31912	4
7	843.40356	871.39848	P	385.25580	3
8	956.48763	984.48255	L	288.20303	2
9			R	175.11896	1

Figure S5. MS and MS/MS spectra of the peptides matched to HDAC1. All raw data generated on LTQ-Orbitrap (Thermo Scientific) were re-processed using Proteome Discoverer software (v1.3, Thermo Scientific), which resulted in one more unique peptide (YYAVNYPLR) of HDAC1 compared to the previous results from Bioworks (v3.3.1SP1) search (Figure S4B). The AACT/SILAC pairs of both peptides and a representative MS/MS are shown.
