# Supplementary material for: 14-3-3ε Mediates the Cell Fate Decision-Making Pathways in Response of Hepatocellular Carcinoma to Bleomycin-Induced DNA Damage
Source: PLoS One. 2013 Mar 5;8(3):e55268. doi: 10.1371/journal.pone.0055268 (PMC3589417; doi:10.1371/journal.pone.0055268)
Supplement: Figure S6 — Ser412 on TAK1 was identified as a BLM-induced phosphorylation site by MS/MS. The MS/MS spectra of both phosphorylated and unphosphorylated sequence (SIQDLTVTGTEPGQVSSR) were shown. (PDF) [file pone.0055268.s006.pdf]

**Figure S6**

SIQDLTVTGTEPGQVSSR
(unphosphorylated form)

| m/z | Charge | ΔM (ppm) | Mascot Ion Score | Sequest Xcorr |
|---|---|---|---|---|
| 937.98 | 2+ | 5.40 | 62 | 4.23 |

| #1 | $b^+$ | $b^{2+}$ | Seq. | $y^+$ | $y^{2+}$ | #2 |
|---|---|---|---|---|---|---|
| 1 | 88.03931 | 44.52329 | S | | | 18 |
| 2 | 201.12338 | 101.06533 | I | 1787.91346 | 894.46037 | 17 |
| 3 | 329.18196 | 165.09462 | Q | 1674.82939 | 837.91833 | 16 |
| 4 | 444.20891 | 222.60809 | D | 1546.77081 | 773.88904 | 15 |
| 5 | 557.29298 | 279.15013 | L | 1431.74386 | 716.37557 | 14 |
| 6 | 658.34066 | 329.67397 | T | 1318.65979 | 659.83353 | 13 |
| 7 | 757.40908 | 379.20818 | V | 1217.61211 | 609.30969 | 12 |
| 8 | 858.45676 | 429.73202 | T | 1118.54369 | 559.77548 | 11 |
| 9 | 915.47823 | 458.24275 | G | 1017.49601 | 509.25164 | 10 |
| 10 | 1016.52591 | 508.76659 | T | 960.47454 | 480.74091 | 9 |
| 11 | 1145.56851 | 573.28789 | E | 859.42686 | 430.21707 | 8 |
| 12 | 1242.62128 | 621.81428 | P | 730.38426 | 365.69577 | 7 |
| 13 | 1299.64275 | 650.32501 | G | 633.33149 | 317.16938 | 6 |
| 14 | 1427.70133 | 714.35430 | Q | 576.31002 | 288.65865 | 5 |
| 15 | 1526.76975 | 763.88851 | V | 448.25144 | 224.62936 | 4 |
| 16 | 1613.80178 | 807.40453 | S | 349.18302 | 175.09515 | 3 |
| 17 | 1700.83381 | 850.92054 | S | 262.15099 | 131.57913 | 2 |
| 18 | | | R | 175.11896 | 88.06312 | 1 |

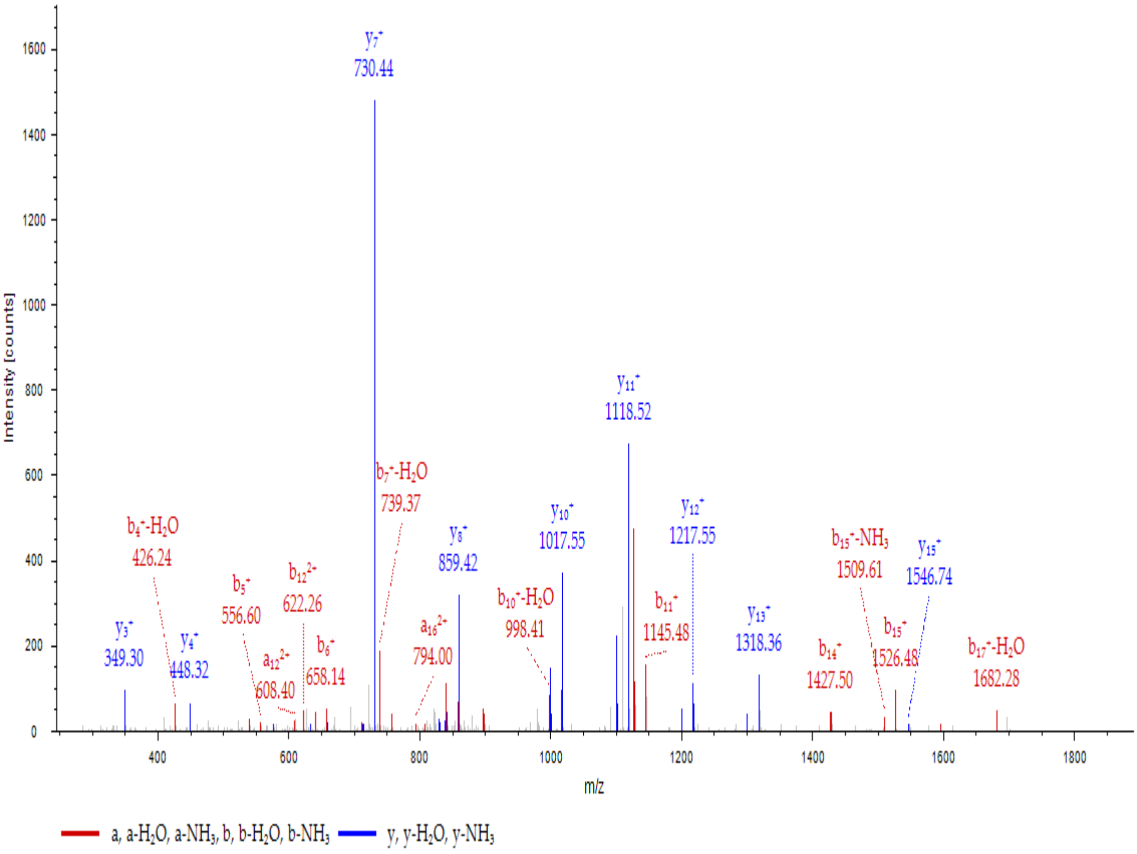

$S_p$IQDLTVTGTEPGQVSSR
(phosphorylated form)

| m/z | Charge | ΔM (ppm) | Mascot Ion Score | Sequest Xcorr |
|---|---|---|---|---|
| 977.96 | 2+ | 4.92 | 57 | 4.73 |

| #1 | $b^+$ | $b^{2+}$ | Seq. | $y^+$ | $y^{2+}$ | #2 |
|---|---|---|---|---|---|---|
| 1 | 168.00564 | 84.50646 | S-Phospho | | | 18 |
| 2 | 281.08971 | 141.04849 | I | 1787.91346 | 894.46037 | 17 |
| 3 | 409.14829 | 205.07778 | Q | 1674.82939 | 837.91833 | 16 |
| 4 | 524.17524 | 262.59126 | D | 1546.77081 | 773.88904 | 15 |
| 5 | 637.25931 | 319.13329 | L | 1431.74386 | 716.37557 | 14 |
| 6 | 738.30699 | 369.65713 | T | 1318.65979 | 659.83353 | 13 |
| 7 | 837.37541 | 419.19134 | V | 1217.61211 | 609.30969 | 12 |
| 8 | 938.42309 | 469.71518 | T | 1118.54369 | 559.77548 | 11 |
| 9 | 995.44456 | 498.22592 | G | 1017.49601 | 509.25164 | 10 |
| 10 | 1096.49224 | 548.74976 | T | 960.47454 | 480.74091 | 9 |
| 11 | 1225.53484 | 613.27106 | E | 859.42686 | 430.21707 | 8 |
| 12 | 1322.58761 | 661.79744 | P | 730.38426 | 365.69577 | 7 |
| 13 | 1379.60908 | 690.30818 | G | 633.33149 | 317.16938 | 6 |
| 14 | 1507.66766 | 754.33747 | Q | 576.31002 | 288.65865 | 5 |
| 15 | 1606.73608 | 803.87168 | V | 448.25144 | 224.62936 | 4 |
| 16 | 1693.76811 | 847.38769 | S | 349.18302 | 175.09515 | 3 |
| 17 | 1780.80014 | 890.90371 | S | 262.15099 | 131.57913 | 2 |
| 18 | | | R | 175.11896 | 88.06312 | 1 |

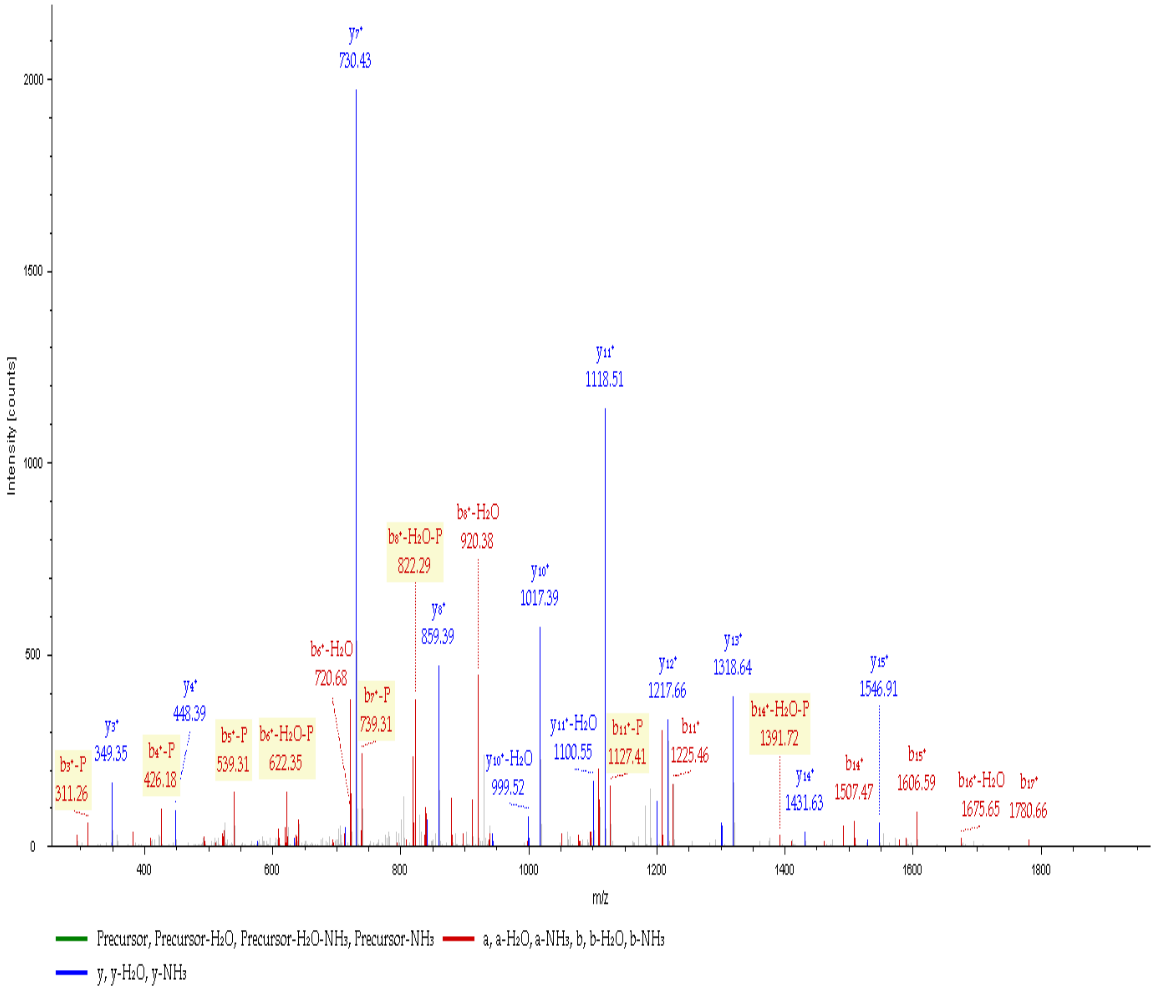


**Figure S6. Ser412 on TAK1 was identified as a BLM-induced phosphorylation site by MS/MS.** The MS/MS spectra of both phosphorylated and unphosphorylated sequence (SIQDLTVTGTEPGQVSSR) were shown.
