# Supplementary material for: 14-3-3ε Mediates the Cell Fate Decision-Making Pathways in Response of Hepatocellular Carcinoma to Bleomycin-Induced DNA Damage
Source: PLoS One. 2013 Mar 5;8(3):e55268. doi: 10.1371/journal.pone.0055268 (PMC3589417; doi:10.1371/journal.pone.0055268)
Supplement: Table S3 — Categorization of the identified BLM-induced 14-3-3ε interactors and their associations with different biological processes or pathways. All of MS-indentified BLM-induced HCC-specific 14-3-3ε interactors were grouped into the specific biological processes (BPs) or pathways by using online database-Gene Ontology (http://geneontology.org/) and DAVID (http://david.abcc.ncifcrf.gov). (PDF) [file pone.0055268.s009.pdf]

Table S3. The identified BLM-induced 14-3-3ε interactors and their associations with different biological processes or pathways

IPI No.	Gene Symbol	Protein Name	Pep. ID (Pep*)[a]	Ratio (L/H)[b]	SD[c]
IPI00000816.1	YWHAE	14-3-3 epsilon (bait)	10(5)	1.00	0.11
		**Chromosome remodeling**			
IPI00005711.2	HDAC6	histone deacetylase 6	2(2)	2.77	0.57
IPI00013774.1	HDAC1	histone deacetylase 1	1(1)	1.97	
IPI00171798.1	MTA2	metastasis associated 1 family, member 2	1(1)	2.19	
IPI00299000.5	PA2G4	proliferation-associated protein 2G4	3(2)	1.41	0.54
IPI00328319.8	RBBP4	retinoblastoma binding protein 4	3(2)	1.56	0.05
IPI00413755.1	TAF4	transcription initiation factor TFIID subunit 4	5(3)	2.19	0.04
IPI00150057.6	SMARCC2	isoform 2 of SWI/SNF-related matrix-associated actin-dependent regulator of chromatin subfamily C member 2	4(3)	2.01	0.11
IPI00221035.4	BTF3	isoform 1 of Transcription factor BTF3	2(2)	1.45	0.15
IPI00741005.4	MGA	MAX gene associated	4(4)	2.11	0.57
		**Intracellular protein transport**			
IPI00001639.2	KPNB1	importin subunit beta-1	4(3)	1.85	0.31
IPI00007401.1	IPO8	Importin-8	4(2)	3.17	0.22
IPI00295857.6	COPA	coatomer subunit alpha	12(6)	1.43	0.35
IPI00298961.3	XPO1	exportin-1	3(2)	1.76	0.91

Table S3 Continued

IPI No.	Gene Symbol	Protein Name	Pep. ID (Pep*)[a]	Ratio (L/H)[b]	SD[c]
		**Intracellular signaling cascade**			
IPI00007277.2	LRRFIP2	isoform 1 of leucine-rich repeat flightless-interacting protein 2	5(4)	2.30	0.43
IPI00014264.5	CENTB2	centaurin-beta-2	16(8)	1.43	0.08
IPI00016639.7	PRKCI	protein kinase C, iota	3(3)	1.75	0.18
IPI00019459.1	MAP3K7IP1	mitogen-activated protein kinase kinase kinase 7-interacting protein 1	11(5)	1.86	0.36
IPI00784013.1	JAK1	janus kinase 1	6(4)	1.80	0.30
IPI00166840.3	MAP3K7IP3	isoform 1 of Mitogen-activated protein kinase kinase kinase 7-interacting protein 3	4(2)	2.63	0.11
IPI00218566.1	MAP3K7	isoform 1A of Mitogen-activated protein kinase kinase kinase 7	6(4)	1.76	0.36
IPI00168691.7	SHCBP1	SHC SH2 domain-binding protein 1	4(3)	2.90	1.45
IPI00237011.5	STK38L	serine/threonine-protein kinase 38-like	13(5)	2.13	0.23
		**Cell cycle**			
IPI00001560.2	CDKN2A	cyclin-dependent kinase inhibitor	3(2)	1.52	0.38
IPI00007927.3	SMC2	isoform 1 of Structural maintenance of chromosomes protein 2	3(1)	2.31	
IPI00022043.1	TSC1	hamartin	2(2)	1.53	0.21
IPI00550655.4	SPIN1	spindlin-1	4(2)	1.71	0.12
		**Apoptosis**			
IPI00001871.2	PAWR	PRKC apoptosis WT1 regulator protein	4(2)	3.25	0.88
IPI00013735.1	FASTKD2	isoform 1 of FAST kinase domain-containing protein 2	2(2)	1.57	0.21
IPI00022542.1	ROCK1	Rho-associated protein kinase 1	14(6)	2.08	0.29

Table S3 Continued

IPI No.	Gene Symbol	Protein Name	Pep. ID (Pep*)[a]	Ratio (L/H)	SD[c]
		**DNA and RNA binding**			
IPI00012442.1	G3BP1	Ras GTPase-activating protein-binding protein 1	2(2)	1.86	0.13
IPI00012726.4	PABPC4	isoform 1 of polyadenylate-binding protein 4	4(1)	1.53	
IPI00304596.3	NONO	Non-POU domain-containing octamer-binding protein	5(3)	1.46	0.19
IPI00017297.1	MATR3	Matrin-3	4(2)	1.41	0.61
IPI00030131.3	TMPO	isoform Beta of lamina-associated polypeptide 2, isoforms beta/gamma	3(2)	1.84	0.05
IPI00030320.4	DDX6	probable ATP-dependent RNA helicase DDX6	2(1)	1.43	
IPI00844578.1	DHX9	ATP-dependent RNA helicase A	9(5)	1.41	0.22
		**RNA splicing**			
IPI00009328.4	EIF4A3	eukaryotic initiation factor 4A-III	4(2)	1.57	0.01
IPI00010740.1	SFPQ	isoform Long of Splicing factor, proline- and glutamine-rich	3(2)	1.43	0.08
IPI00014319.5	IVNS1ABP	influenza virus NS1A-binding protein	10(4)	3.08	0.37
IPI00026089.3	SF3B1	splicing factor 3B subunit 1	7(3)	1.48	0.34
IPI00031556.7	U2AF2	splicing factor U2AF 65 kDa subunit	2(2)	1.47	0.50
IPI00221106.5	SF3B2	splicing factor 3B subunit 2	5(2)	1.65	0.30
		**Nucleoside metabolic process**			
IPI00003168.1	PRPSAP2	phosphoribosyl pyrophosphate synthetase-associated protein 2	15(4)	4.06	0.28
IPI00219617.5	PRPS2	isoform 1 of Ribose-phosphate pyrophosphokinase 2	3(1)	4.05	
IPI00291578.7	PRPSAP1	phosphoribosyl pyrophosphate synthetase-associated protein 1	9(3)	2.58	0.25
IPI00301263.2	CAD	CAD protein	13(5)	1.48	0.31
IPI00029054.1	NT5C2	cytosolic purine 5'-nucleotidase	2(2)	1.63	0.08

Table S3 Continued

IPI No.	Gene Symbol	Protein Name	Pep. ID (Pep*)[a]	Ratio (L/H)[b]	SD[c]
		**Ubiquitination and ubiquitin cycle**			
IPI00030915.1	USP8	ubiquitin carboxyl-terminal hydrolase 8	5(3)	1.62	0.06
IPI00033130.3	SAE1	SUMO-activating enzyme subunit 1	2(1)	1.53	
IPI00100160.3	CAND1	isoform 1 of Cullin-associated NEDD8-dissociated protein 1	8(4)	1.48	0.29
IPI00293464.5	DDB1	DNA damage-binding protein 1	8(4)	1.44	0.19
IPI00291802.3	LMO7	isoform 3 of LIM domain only protein 7	20(7)	2.86	0.71
IPI00217407.2	UBR2	isoform 4 of E3 ubiquitin-protein ligase UBR2	7(3)	2.54	0.41
IPI00018971.8	TRIM21	52 kDa Ro protein	4(3)	1.79	0.30
IPI00181396.5	VPRBP	isoform 3 of Protein VPRBP	2(2)	1.79	0.29
		**Cytoskeleton and cell motility**			
IPI00013808.1	ACTN4	alpha-actinin-4	16(7)	1.55	0.11
IPI00014516.1	CALD1	isoform 1 of Caldesmon	8(4)	1.43	0.25
IPI00183002.6	PPP1R12A	isoform 1 of Protein phosphatase 1 regulatory subunit 12A	5(4)	1.47	0.33
IPI00005087.1	TMOD3	tropomodulin-3	9(5)	2.53	0.23
		**Translation**			
IPI00022648.2	EIF5	eukaryotic translation initiation factor 5	4(1)	1.76	
IPI00029012.1	EIF3A	eukaryotic translation initiation factor 3 subunit A	4(4)	1.56	0.24
IPI00221300.2	EIF2B1	translation initiation factor eIF-2B subunit alpha	3(3)	1.73	0.36
		**Others**			
IPI00060181.1	EFHD2	EF-hand domain-containing protein D2	2(2)	2.66	0.03
IPI00220249.3	LTBP1	latent-transforming growth factor beta-binding protein, isoform 1L precursor	5(1)	1.70	
IPI00305267.2	GOLGA3	isoform 1 of Golgin subfamily A member 3	28(8)	1.47	0.40

Table S3. Categorization of the identified BLM-induced 14-3-3ε interactors and their associations with different biological processes or pathways. All of MS-indentified

BLM-induced HCC-specific 14-3-3ε interactors were grouped into the specific biological processes (BPs) or pathways by using online database-Gene Ontology (http://geneontology.org/) and DAVID (http://david.abcc.ncifcrf.gov).

a: Pep. ID refers to peptide number matched to a specific protein, Pep* refers to peptide number used to quantify the protein. Protein ratio (L/H) was calculated by averaging the ratios of all leucine-containing peptides sequenced for each protein (b), and standard deviation (SD) was given to evaluate the accuracy of protein ratio(c).
