# Supplementary material for: 14-3-3ε Mediates the Cell Fate Decision-Making Pathways in Response of Hepatocellular Carcinoma to Bleomycin-Induced DNA Damage
Source: PLoS One. 2013 Mar 5;8(3):e55268. doi: 10.1371/journal.pone.0055268 (PMC3589417; doi:10.1371/journal.pone.0055268)
Supplement: Table S4 — The list of TAK1 sequence and primers for constructing the expressing plasmids. (PDF) [file pone.0055268.s010.pdf]

Table S4. The list of TAK1 sequence and primers for constructing the expressing plasmids.

1. TAK1(MAP3K7, isoform 1A) protein sequence

(O43318 (M3K7_HUMAN), UniProtKB/Swiss-Prot)

```
        10         20         30         40         50         60
MSTASAASSS SSSSAGEMIE APSQVLNFEE IDYKEIEVEE VVGRGAFGVV CKAKWRAKDV
        70         80         90        100        110        120
AIKQIESESE RKAFIVELRQ LSRVNHPNIV KLYGACLNPV CLVMEYAEGG SLYNVLHGAE
       130        140        150        160        170        180
PLPYYTAAHA MSWCLQCSQG VAYLHSMQPK ALIHRDLKPP NLLLVAGGTV LKICDFGTAC
       190        200        210        220        230        240
DIQTHMTNNK GSAAWMAPEV FEGSNYSEKC DVFSWGIILW EVITRRKPFD EIGGPAFRIM
       250        260        270        280        290        300
WAVHNGTRPP LIKNLPKPIE SLMTRCWSKD PSQRPSMEEI VKIMTHLMRY FPGADEPLQY
       310        320        330        340        350        360
PCQYSDEGQS NSATSTGSFM DIASTNTSNK SDTNMEQVPA TNDTIKRLES KLLKNQAKQQ
       370        380        390        400        410        420
SESGRLSLGA SRGSSVESLP PTSEGKRMSA DMSEIEARIA ATTGNGQPRR RSIQDLTVTG
       430        440        450        460        470        480
TEPGQVSSRS SSPSVRMITT SGPTSEKPTR SHPWTPDDST DTNGSDNSIP MAYLTLDHQL
       490        500        510        520        530        540
QPLAPCPNSK ESMAVFEQHC KMAQEYMKVQ TEIALLLQRK QELVAELDQD EKDQQNTSRL
       550        560        570
VQEHKKLLDE NKSLSTYYQQ CKKQLEVIRS QQQKRQGTS
```

*Residues marked in red were all mutated to **Ala** for the following experiments.

*Residues underlined surrounding Ser268 and Ser432 are putative 14-3-3 binding motifs on TAK1, respectively.

2. Primers for plasmid constructs:

(1) Primers for N-terminal FLAG-tagged 14-3-3ε (NM_006761):

5'-CACCATGGACTACAAGGACGACGATGACAAGGATGATCGAGAGGATCTGGTGT-3'/5'-GTCAGATTTGGTGGTTCTCAGTGAC-3'

The bases underlined represent start codon and FLAG epitope-coded sequence. PCR products were directly introduced into pcDNA™3.1 Directional TOPO Expression vector (Invitrogen, K4900-40) according to the manufacturer's protocol.

(2) Primers for HA-tagged TAB1 (MAP3K3IP1, NM_006116):

5'-ATAATAGTCGACCATGGCGGCGCAGAGGAGGAGCT-3'/5'-TGTAGGTACCCTACGGTGCTGTCACCACGCT-3'

PCR products were introduced into pCMV-HA vector (Clontech) between SalI and KpnI sites.

(3) Primers for HA-tagged full length TAK1 (NM_003188, 1-579AA), TAK1 kinase domain (TAK1-N, 1-300AA) and TAK1-C-terminal domain (TAK1-C, 301-579AA):

5'-ATTAGTCGACCATGTCTACAGCCTCTGCCGCCT-3'/5'-CTAGGGTACCTCATGAAGTGCCTTGTCGT-3'

5'-ATTAGTCGACCATGTCTACAGCCTCTGCCGCCT-3'/5'-GGCCGGTACCTCAATACTGTAATGGCTCATCTG-3'

5'-ACGCGTCGACCCCTTGTCAGTATTCAGATGA-3'/5'-CTAGGGTACCTCATGAAGTGCCTTGTCGT-3'

PCR products of those genes were introduced into pCMV-HA vector (Clontech) between SalI and KpnI sites.

(4) Primers for individual site-specific mutant of HA-tagged TAK1:

HA-TAK1-T184A:

5'-GCCTGTGACATTCAGGCACACATGACCAATAACAAGG-3'/5'-CCTTGTTATTGGTCATGTGTGCCTGAATGTCACAGGC-3'

HA-TAK1-T187A:

5'-CAGACACACATGGCCAATAACAAGGGGAGTGCTGC-3'/5'-GCAGCACTCCCCTTGTTATTGGCCATGTGTGTCTG-3'

HA-TAK1-S192A:

5'-CCAATAACAAGGGGGCTGCTGCTTGGATGGC-3'/5'-GCCATCCAAGCAGCAGCCCCCTTGTTATTGG-3'

HA-TAK1-S268A:

5'-GATGACTCGTTGTTGGGCTAAAGATCCTTCCCAGCGCC-3'/5'-GGCGCTGGGAAGGATCTTTAGCCCAACAACGAGTCATC-3'

HA-TAK1-S412A:

5'-GGACAGCCAAGACGTAGAGCCATCCAAGACTTGACTGTAACTGG-3'/5'-CCAGTTACAGTCAAGTCTTGGATGGCTCTACGTCTTGGCTGTCC-3'

HA-TAK1-S432A:

5'-TGAGCAGTAGGTCATCCGCTCCCAGTGTCAGAATGATTACTACC-3'/5'-GGTAG TAATCATTCTGACACTGGGAGCGGATGACCTACTGCTCAC-3'

Using pHA-TAK1 as template, all mutants were generated according to site-directed mutagenesis method.

All insert constructs listed above were confirmed by DNA sequencing.
